# Defining ‘Abortion’: a call for clarity

**DOI:** 10.1007/s11017-025-09706-5

**Published:** 2025-03-22

**Authors:** Nicholas Colgrove

**Affiliations:** Augusta University, 2500 Walton Way, Augusta, GA 30904 USA

**Keywords:** Abortion, Women’s rights, Pregnancy, Statutory law, Ectopic pregnancy

## Abstract

In *Dobbs v. Jackson*, the Supreme Court found that ‘the Constitution does not confer a right to abortion.’ Rather, individual states must determine whether a right to abortion exists. Following *Dobbs*, state abortion laws have diverged significantly. This has generated confusion over what the law permits. Consequently, some pregnant women reportedly have not received timely treatment for life-threatening conditions. Clear guidance on abortion policy is essential, therefore, since continued confusion risks lives. Sweeping calls to improve patient access to abortion will not provide clear guidance, however, since ‘abortion’ is defined differently across jurisdictions. In fact, there are six variables to consider when defining ‘abortion’: (1) the definition of ‘pregnancy,’ (2) whether prescribing abortifacients counts as an abortion, (3) whether abortion successfully terminates pregnancy, (4) whether abortion has some characteristic intention, (5) whether providers must know that they likely will harm fetuses, and (6) whether providers must know that their patients are pregnant. States address each variable differently, so ‘abortion’ means different things across jurisdictions. One may respond that legislators are solely to blame for confusion here, since medical experts, by contrast, possesses a clear definition of ‘abortion.’ Not so. ‘Abortion’ is defined inconsistently throughout the medical literature too. As such, both legal and medical domains would benefit from careful discussions of ‘abortion.’ Attending to the six variables identified here is a good starting place. In this essay, I suggest how best to think about each and propose a definition of ‘abortion’ well-suited for developing clear abortion policy in a polarized society.

## Introduction

In *Dobbs v. Jackson*, the Supreme Court of the United States ruled that “the Constitution does not confer a right to abortion” [1]. Instead, whether there is a right to abortion must be determined by “the people and their elected representatives” [1]. Following *Dobbs*, U.S. state laws on abortion have changed rapidly and diverged significantly. Amidst these changes, confusion over what is allowed under the law has led to reports of pregnant women with serious and life-threatening conditions being denied timely treatment. For example, Carpenter et al. lament that post-*Dobbs*, “there are more cases of individuals denied abortion despite fetal demise or lethal fetal abnormality” [2, p. 310]. Arey et al. state that one provider in Texas “reported that their hospital no longer offers treatment for ectopic pregnancies implanted in cesarean scars” [3, p. 389]. Minkoff, Vullikanti, and Marshall generalize on this claim, stating that “Texas hospitals no longer offer treatment for ectopic pregnancies” [4, p. 15]. They also argue that anti-abortion policy “endangers pregnant people who undergo any medical procedure that can harm a fertilized egg” [4, p. 15]. Prescriptions for “immune and inflammatory disorders,” for example, may be withheld given these drugs’ “teratogenic effects on the fetus” [4, p. 15]. Nambiar et al. add that maternal morbidity seemingly runs higher in states with restrictive abortion laws [5]. Reportedly, “57% of patients” in their study had “a serious maternal morbidity compared with 33% who elected immediate pregnancy interruption under similar clinical circumstances reported in states without such [restrictive] legislation” [5, p. 649]. So, changes to abortion restrictions not only affect abortion access but access to other interventions as well which may, in turn, place women at grave risk of harm.

Some concerns may be exaggerated. Contra Minkoff, Vullikanti, and Marshall, I have argued that Texas’s statutes define “abortion” in a way that does not apply to treatment for ectopic pregnancies generally [6, p. 201]. That there is significant confusion among scholars and clinicians over what is permitted, however, illustrates a need for clarity in “the writing, revising, understanding, and applying of the law” [6, p. 202]. Unfortunately, what state laws say is often addressed in a piecemeal manner only. This occurs in recent work by Hersey, Potter-Rutledge, and Brown [7]—as well as Minkoff, Vullikanti, and Marshall [4]—where quotations from a few states’ statutes are isolated because they serve the authors’ purposes. Clarity will be best served by taking a wider look at differences between states’ laws, however. Failure to do so overlooks two problems.

First, with respect to abortion and reproductive care, calls for uniformity in policy across jurisdictions face substantial obstacles. This is not merely due to disagreement over the permissibility of abortion. It is also because there is no consensus in U.S. statutory law on what terms like ‘abortion’ mean. Not only do different jurisdictions draw different conclusions as to whether abortion is necessary (or permissible) in a given case; they also disagree on whether an abortion is even *performed* in many cases. When scholars discuss laws “that seek to make abortion illegal,” [8, p. 952] therefore, they must recognize that the referent of ‘abortion’ varies across jurisdictions, so interventions prohibited by different states’ abortion restrictions vary as well.

Second, given substantial variation across jurisdictions in terms like ‘abortion,’ statements from major medical organizations—like the American College of Obstetricians and Gynecologists, which claims “abortion is a safe, essential part of comprehensive health care”—will mean different things in different places [9]. This is not to deny that ‘abortion is a safe, essential part’ of care. I take no stance on the matter here. Rather, if the meaning of ‘abortion’ is highly ambiguous, then it is unclear which *particular* procedures are being referred to as ‘safe’ and ‘essential’ in a given jurisdiction.^1^ As one example, consider the claim that ‘abortion is required to treat ectopic pregnancy.’ This is not regarded as true in every jurisdiction, even granting that *some* intervention is necessary to treat ectopic pregnancy. Thus, treatment recommendations that are intended to apply across jurisdictions—including federal recommendations—are unlikely to provide clear, effective guidance (that is, until some universally accepted–or universally enforced–definitions for terms like ‘abortion’ are employed). Absent clear guidance, and while widespread confusion over abortion law persists, the health and lives of many pregnant women remain at stake.

I will address these concerns, beginning with a description of the major differences between U.S. states regarding how terms like ‘abortion’ are defined in statutory law. States almost universally assert that abortion involves ‘termination of pregnancy.’ Any real consensus is illusory, however, since ‘pregnancy’ and ‘termination’ are often defined differently. The phrase, ‘termination of pregnancy’, therefore, means different things in different jurisdictions, legally speaking.

Looking closely at the statutory language, there are six variables on which state statutes often diverge. These variables generate six questions to consider when defining ‘abortion’ and related terms in public policy (whether in the U.S. or beyond):

1. What is the definition of ‘pregnancy’ (i.e., what is the thing being terminated)?
2. Does prescribing an abortion-inducing medication count as performing an abortion?
3. Does abortion terminate pregnancy or is it merely an act performed with ‘the intention to terminate pregnancy’?
4. Aside from an ‘intention to terminate pregnancy,’ does abortion have any other characteristic intention(s)?
5. Must a provider know that their action is likely to cause embryonic or fetal death?
6. Must a provider know that the person receiving an abortion is pregnant?

In Sect. "Six points of disagreement in U.S. statutory law", I document how U.S. states (henceforth, ‘states’) each answer these questions in statutory law. I also consider the possible rationale behind (and implications for) different answers.

In Sect. Are legislators the problem?, I consider and respond to a major objection: that *legislators*, especially anti-abortion legislators, are solely to blame for confusion over ‘abortion.’ Donley and Kelly suggest this, asserting that, by introducing inconsistencies into legal definitions of ‘abortion,’ “antiabortion legislatures are running a fool’s errand” since “it is impossible to fully distinguish abortion from other types of reproductive healthcare” [10, p. 1]. Rather than seek to define ‘abortion’ on their own, Donley and Kelly suggest that legislators should follow the medical community’s lead (e.g., perhaps by codifying the ‘medical definition’ of ‘abortion’ into statutory law) [10, pp. 5–6]. In response, the medical domain does not fare much better than the legal domain when it comes to providing a clear, consistent definition of ‘abortion.’ There exists no canonical ‘medical definition’ of ‘abortion.’ Confusion and ambiguity, therefore, are not isolated to the legal domain; they infect the medical domain too. Thus, in its *present* state, the medical community is also not well-positioned to resolve confusion over ‘abortion.’ Hence, both legal and medical domains stand to benefit from careful, rigorous discussions of how best to define ‘abortion.’

Since inconsistent definitions of ‘abortion’ pervade both the legal and medical domains, in Sect. Answering the six questions, I suggest answers to each of the six questions (listed above) that might facilitate the development of clear abortion policy in a polarized society. My intent is not to provide ‘the right answers.’ Rather, I defend *reasonable* answers for the specific purpose of developing clear policy. How best to answer each of these questions remains open for debate as does the question of *who* should answer each question (whether legislators, medical experts, philosophers, etc.). For this reason, my suggested answers should be read as initiating a conversation, not settling one.

Finally, in Sect. A proposed definition of ‘abortion’, I use my proposed answers to formulate a definition of ‘abortion.’ I argue that a definition of ‘abortion’ should align with reasonable answers to all six questions and that, in polarized society, the definition should also limit its referents to exactly the cases that are in dispute (insofar as is possible). Such a definition would enable legislators to provide maximum clarity to clinicians (in terms of what is permitted) in cases of pregnancy termination where there are no real disputes. Furthermore, if one defines ‘abortion’ in this way, then abortion bans in restrictive states would prohibit the fewest number of practices possible (i.e., only those practices that are regarded as impermissible by a nonnegligible portion of the population). Limiting abortion restrictions to the fewest number of cases possible should appeal to abortion advocates, since it would ensure the greatest amount of reproductive autonomy in restrictive states. Restricting access to all and only the practices that are in dispute should also appeal to abortion opponents, since the law would still prohibit practices that they find objectionable. And achieving maximum clarity in policy—which minimizes unnecessary harm that results from *confusion*—should appeal to everyone.

As one last qualifier, readers may disagree with my suggested answers and proposed definition of ‘abortion.’ By identifying six major sources of disagreement over ‘abortion,’ however, this essay provides a solid framework to rely upon when crafting clear and informative policies on abortion. This framework is fully applicable to jurisdictions outside of the U.S. and may be used to bring clarity to legal, medical, and academic discussions of abortion. For any abortion law, policy, statement, or argument, the same six questions must be answered in the interest of defining ‘abortion’ clearly. Clear guidance obviously matters since—aside from ensuring that experts are not talking past one another—the above reports indicate that confusion over abortion policy puts pregnant women at risk of serious harm and death. Thus, there are compelling reasons to work towards resolving confusion. This essay takes a major step towards doing so.

## Six points of disagreement in U.S. statutory law

I begin by documenting how each state within the U.S. responds to each of the six questions listed above. Obviously, there already exist many summaries of U.S. abortion law. Why should another summary matter? Because identifying differences in the *definition* of ‘abortion’ is critical when comparing states’ laws. When ‘abortion’ is defined differently in each state, two states may appear to restrict abortion in the same way, but given different definitions of ‘abortion,’ what is restricted actually differs. Thus, *effective* comparisons of state laws requires that one *first* attend to how each state defines ‘abortion.’ Relatedly, consider frequently-cited polls—like the Gallup Poll—that describe Americans’ attitudes toward abortion [11]. I establish that there is no real consistency in how ‘abortion’ is defined across jurisdictions (i.e., among legal experts). Nor is there consistency among medical experts. If so, then it seems absurd to think that laypeople possess anything like a clear, consistent definition of ‘abortion’ when responding to polls and surveys. Thus, whether one is comparing state laws or surveying the public on their attitudes towards abortion, this research would be *far* more meaningful if one begins by getting clear on what ‘abortion’ means. This is one major feature of my analysis that sets it apart from others (and, in that regard, it provides the basis for more meaningful empirical research on abortion).^2^

### Competing definitions of ‘pregnancy’

Nearly every state defines abortion as involving ‘termination of pregnancy.’ The meaning of this phrase varies, however, since ‘pregnancy’ is often defined differently (see Table 1).^3^

**Table 1:** Definitions of ‘pregnancy’

How does each state define ‘pregnancy’?	
Pregnancy begins at conception. (*n* = 18)	AL, AR, AZ, FL, ID, KS, KY, LA, MS, MT, NE, OH, OK, PA, SC, TN, TX, WY
Pregnancy begins at implantation. (*n* = 7)	CA, CO, IL, MA, NH, WA, WV
Not explicitly defined in statutory law. (*n* = 25)	AK, CT, DE, GA, HI, IN, IA, MD, ME, MI, MN, MO, NC, ND, NJ, NM, NV, NY, OR, RI, SD, UT, VA, VT, WI

Eighteen states define pregnancy as beginning at conception (or fertilization). Seven states assert that pregnancy begins with implantation (namely, when an embryo attaches itself to the uterine wall). Conception and implantation are two distinct biological events, however. Conception always precedes implantation by around six days [12]. This means that legally speaking, someone may count as pregnant in one state but not pregnant in others.

How ‘pregnancy’ is defined affects policy on contraceptives [13]. Contraceptives, by definition, prevent pregnancy from occurring. In principle, this means that interventions that allow conception but prevent implantation—including certain intrauterine devices [12]—may count as contraceptives in some states and non-contraceptives in others. For any intervention known to prevent implantation, therefore, access to that intervention may be restricted in states that outlaw abortion. To those who believe pregnancy begins at implantation—who subsequently believe that relevant interventions are contraceptives—this may look like legislative overreach rooted in “bad science” [14]. Worse, for interventions that *may* prevent implantation, states that claim pregnancy begins at conception might regard these interventions as abortifacients and restrict their use accordingly.

To complicate matters, some states that claim pregnancy begins at conception also explicitly define anti-implantation methods as ‘contraceptives’ nonetheless. See, for example, Arizona [15], Idaho [16], Kansas [17], and South Carolina [18]. In these states, interventions that (by definition) interrupt pregnancy are classified as ‘contraceptives’ (which, by definition, prevent pregnancy). So, not only does the meaning of terms like ‘pregnancy’ and ‘contraceptive’ vary across jurisdictions, but some states define terms inconsistently. Subsequent confusion over which interventions are legally permitted is therefore unsurprising.

### ‘Termination’ as performing, prescribing, or both?

‘Pregnancy’ aside, if abortion involves ‘termination of pregnancy,’ what does ‘termination’ mean? *Methods* of termination often include medication, surgical devices, suction, etc. On this point, state laws usually align: the method of termination is not usually considered relevant to whether an act counts as an abortion or not. Where states differ is regarding acts of prescribing (see Table 2).

**Table 2:** Acts of prescribing

Does ‘abortion’ include acts of *prescribing* an abortive means (explicitly)?	
Yes. (*n* = 17)	AK, AL, AR, GA, KS, LA, MN, MO, MS, MT, ND, NE, NH, OK, SC, TX, UT
No. (*n* = 29)	AZ, CA, CO, CT, DE, FL, HI, IL, IN, IA, KY, MA, MD, ME, MI, NM, NJ, NV, NY, OH, OR, PA, RI, SD, VA, VT, WA, WI, WV
Both of the above.^1^ (*n* = 4)	ID, NC, TN, WY

Four states refer to prescribing as an abortion in one statute, while omitting this language in other statutes

If merely prescribing an abortifacient is the same as performing an abortion, then concerns raised in the previous section—regarding what counts as a contraceptive—are compounded. Prescribers must not only understand how ‘pregnancy’ and ‘contraceptive’ are defined within their jurisdiction, but they must also know whether they perform an abortion simply by prescribing a drug. Subsequently, it is unsurprising if some providers are hesitant to prescribe drugs that *may* count as an abortifacient, especially when doing so might amount to performing an abortion, legally speaking.

If prescribing does *not* constitute performing an abortion, then states with restrictive abortion laws are in a difficult position. Historically, anti-abortion laws aimed primarily to penalize abortion providers rather than patients [19–21]. With self-administered prescriptions, however, patients seemingly become the providers. Laws that currently penalize providers, therefore, would seemingly have to penalize patients directly [22]. For restrictive states that wish to penalize medical providers rather than patients, therefore, identifying acts of *prescribing* as ‘abortions’ offers a solution: prescribers are penalized, but patients who self-administer abortifacients are not. Still, doing so is confusing, because it blurs an intuitive distinction between *performing* an abortion and *enabling* the performance of an abortion.^4^

### Must abortion actually terminate pregnancy?

Next, states disagree over whether an abortion is an act that terminates pregnancy or merely an act performed ‘with the intention to terminate pregnancy’ (see Table 3).

**Table 3:** On termination. Insufficient information in statutory law (*n* = 2): MD, NY

Does abortion require the actual termination of pregnancy?	
Yes, abortion is an act that ‘terminates pregnancy.’ (*n* = 22)	AK, CO, CT, DE, FL, HI, IL, IN, IA, KS, ME, MI, MN, NC, NJ, NM, NV, OH, OR, PA, SD, VA
No, abortion is an act that is done ‘with intent to terminate pregnancy.’ (*n* = 11)	AL, CA, GA, MA, RI, TN, TX, UT, VT, WI, WV
Both of the above. (*n* = 15)	AR, AZ, ID, KY, LA, MO, MS, MT, ND, NE, NH, OK, SC, WA, WY

Acts that terminate pregnancy differ from acts performed with an intention to terminate pregnancy. After all, actions done ‘with the intention to terminate’ may fail. Similarly, pregnancy may be terminated without an agent intending that termination occur. As a result, these distinctions generate four categories of actions to consider, *any of which* could be a referent of ‘abortion’:

1. Actions done with the intention to terminate pregnancy that terminate pregnancy,
2. Actions done with the intention to terminate pregnancy that do not terminate pregnancy,
3. Actions done with no intention to terminate pregnancy that terminate pregnancy, and
4. Actions done with no intention to terminate pregnancy that do not terminate pregnancy.

In 11 states, abortion is an action performed with the intention to terminate pregnancy. In these jurisdictions, some actions in categories 1 and 2 are the referent of ‘abortion.’ In 22 states, where abortion is an action that terminates pregnancy, some actions in categories 1 and 3 are the referent of ‘abortion.’ In 15 states, sometimes ‘abortion’ refers to categories 1 and 2, but other times, ‘abortion’ refers to categories 1 and 3. Within these states, therefore, the meaning of ‘abortion’ varies by context. Fortunately, there is *some* overlap here. Across jurisdictions, actions in category 1 are generally regarded as abortions and actions in category 4 are generally regarded as non-abortive. Inconsistencies on categories 2 and 3, however, remain a potential source of confusion.

Worse, ‘termination’ is ambiguous in another way, not reflected by Table 3. ‘Termination’ may refer to an act that transitions a pregnant individual to a state of non-pregnancy *or* it may refer to an act that causes the death of an embryo or fetus [23, 24]. Accounting for this ambiguity doubles the number of categories to which ‘abortion’ might refer. Category 1, for instance, would split into two forms:

1a. Actions done with the intention to transition a pregnant individual to a state of non-pregnancy that do so, or

1b﻿. Actions done with the intention to cause the death of an embryo or fetus that do so.

Depending on how one defines ‘termination,’ therefore, ‘abortion’ will refer to different sets of actions. Suppose, for example, that someone seeks an abortion and, following the intervention, her fetus is born alive. States that rely on 1a may refer to the provider’s action as an abortion. States that rely on 1b may describe that action as an *attempted* abortion only (since the fetus did not die).

Similarly, when a salpingostomy is performed to remove a tubal ectopic pregnancy—where an incision is made into the fallopian tube and the embryo within it is extracted, intact—states relying on 1a may regard this action as an abortion. States relying on 1b may not, since performing a salpingostomy in this way need not carry an intention to cause the embryo’s death [25, 26]. Thus, the same procedure may count as an ‘abortion’ in some states, but not in others.

### Identifying the characteristic intention (or purpose) behind an abortion

Whether an act ‘terminates pregnancy’ or is merely performed ‘with the intention to terminate pregnancy,’ these features are *still* not enough to distinguish abortions from non-abortions clearly. After all, every induced vaginal delivery and every cesarean section is performed with an intention to terminate pregnancy in the sense described by 1a, above. But no state regards all deliveries as ‘abortions.’ To say otherwise would be to overgeneralize on ‘abortion.’

To avoid overgeneralization, state statutes typically structure language in one of two ways. Some states assert that, by definition, abortion involves having a specific (characteristic) intention, such as ‘killing the embryo or fetus.’ If so, then presumably, acts performed without this intention are not abortions. Alternatively, most states assert that abortion is an act performed with any intention (or purpose) ‘other than’ those on a specified list. For example, many states assert that ‘abortion’ refers to acts that terminate pregnancy done with any intention *other than* ‘treatment of ectopic pregnancy,’ ‘preserving the health of the fetus,’ and so forth (see Table 4 for an overview).^5^

**Table 4:** Characteristic intention(s). Insufficient information in statutory law (*n* = 4): HI, MD, NY, VA

Does abortion involve termination of pregnancy with some *specific* intention/purpose; or does abortion involve termination with any intention/purpose ‘*other than*’ some specified intentions/purposes?	
Abortion involves termination with some *specific* intention (or purpose), namely ‘intent to cause fetal death’ or ‘to cause the death of the unborn child.’ (*n* = 11)	KY, MS, NE, OK, SC, SD, TX, UT, WI, WV, WY
Abortion involves termination with *any* intention (or purpose) *other than* the intention to produce a live birth or increase the chances of one. (*n* = 37)	AK, AL, AZ, CA, CO, CT, DE, FL, GA, ID, IL, IN, IA, KS, KY, LA, MA, ME, MI, MO, MS, MT, NC, ND, NE, NH, NM, NV, OH, OK, OR, SC, TN, VT, WA, WI, WV
Abortion involves termination with *any* intention (or purpose) *other than* the intention to preserve the life and health of the fetus (or newborn). (*n* = 24)	AK, AL, AR, AZ, DE, GA, ID, IL, KS, KY, LA, MI, MS, MT, NC, ND, NE, NH, OK, SC, TN, TX, WI, WY
Abortion involves termination with *any* intention (or purpose) *other than* the intention to remove an ectopic pregnancy or a molar pregnancy. (*n* = 19)	AL, AR, AZ, GA, ID, KS, LA, MI, MS, MT, NC, ND, NE, NH, OK, TN, TX, UT, WY
Abortion involves termination with *any* intention (or purpose) *other than* the intention to remove fetal remains (without further qualification). (*n* = 25)	AK, AL, AZ, DE, FL, ID, IL, IN, IA, KY, ME, MI, MO, MS, MT, ND, NE, NV, OH, OK, OR, SC, TN, UT, WI
Abortion involves termination with *any* intention (or purpose) *other than* the intention to remove fetal remains where fetal death occurred naturally. (*n* = 20)	AL, AR, GA, ID, IA, KS, LA, MA, MI, NC, ND, NE, NH, OK, RI, SD, TN, TX, WV, WY
Abortion involves termination with *any* intention (or purpose) *other than* the intention to remove fetal remains where fetal death occurred accidentally. (*n* = 9)	AL, AR, ID, KS, LA, MI, NC, NE, OK
Abortion involves termination with *any* intention (or purpose) *other than* the intention to remove fetal remains where fetal death occurred following criminal assault. (*n* = 9)	AL, AR, ID, KS, LA, MI, NC, NE, OK
Abortion involves termination with *any* intention (or purpose) *other than* the intention to treat the mother for an illness or disease, where treatment has an abortive side effect. (*n* = 11)	AL, AR, AZ, IA, LA, MI, MS, MT, OK, TN, WY

If ‘abortion’ refers only to acts performed with a specific intention, then the referent of ‘abortion’ is relatively narrow. In principle, under this definition, someone who self-administers an abortifacient while intending ‘only not to be pregnant’ does not perform an abortion.

Alternatively, if ‘abortion’ refers to any termination performed *without* certain intentions, then the term refers to many more phenomena. On this view, someone who self-administers an abortifacient while intending ‘only not to be pregnant’ *would* likely perform an abortion. Still, on this second approach, not every termination counts as an abortion. In 19 states, termination performed with an intention to treat ectopic pregnancy is not an abortion, legally speaking. So, any abortion ban in these states will not prohibit treatment for ectopic pregnancy. To clarify, treatment for ectopic pregnancy in these 19 states is not permitted because it is regarded as a ‘medically necessary abortion.’ Rather, treatment for ectopic pregnancy is permitted because it is not considered an abortion in the first place.

Regarding how intentions (allegedly) distinguish abortions from non-abortions, states often provide conflicting answers. One Kentucky statute, for example, defines ‘abortion’ as an action performed “with intent to cause fetal death” [29]. This is why it appears in row 1 of Table 4. When discussing abortion performed on minors, however, a different Kentucky statute defines ‘abortion’ as an action done “with intent other than to increase the probability of a live birth … or to remove a dead fetus” [30]. Hence, Kentucky also appears in rows 2 and 5 of Table 4. So, within one state, sometimes abortion is said to involve having a specific intention and sometimes abortion is said to involve having any intention *other than* those specified.

There is nothing inherently wrong with states and scholars defining the same term differently in different contexts. But here, doing so risks exacerbating confusion over abortion policy. As Table 4 shows, states usually provide contextualized (if not conflicting) answers to the fourth question. Accordingly, confusion among clinicians, scholars, and the public is to be expected.

### Knowledge of potential harms

Intentions aside, states often consider providers’ knowledge when distinguishing abortion from non-abortive acts. To illustrate why, suppose a clinician terminates a pregnancy at 12-weeks’ gestation while truthfully stating that her sole intention was to produce a live birth. If intention were all that mattered, this might preclude her action from counting as an abortion, legally speaking. However, this is counterintuitive.

One solution is to claim the clinician *should have known better*. Namely, she should have known that fetuses cannot survive if pregnancy is terminated at 12-weeks’ gestation. That providers know (or should know) their actions threaten embryos or fetuses, therefore, is factored into some states’ definitions of ‘abortion’ explicitly (see Table 5).

**Table 5:** Knowledge of harm

Does performing an abortion explicitly require knowledge that the intervention is reasonably likely to cause the death of an embryo or fetus?	
Yes, providers must act ‘with knowledge’ that their action ‘will, with reasonable likelihood, cause the death of’ the embryo, fetus, or ‘unborn child.’ (*n* = 12)	AL, AR, AZ, GA, ID, KS, LA, MN, NE, NH, NJ, PA
No. (*n* = 31)	AK, CA, CT, DE, FL, HI, IL, IN, IA, KY, MA, MD, ME, MI, MO, MS, NC, NM, NV, NY, OH, OR, RI, SD, TX, UT, VA, VT, WA, WI, WV
Both of the above. (*n* = 7)	CO, MT, ND, OK, SC, TN, WY

In 31 states, ‘abortion’ is defined without reference to providers’ knowledge of the danger their actions likely pose to embryos or fetuses. In those states, someone may terminate pregnancy—not knowing it will likely cause fetal death—and still perform an abortion.

For 19 states—which do build a knowledge requirement into their definitions—ignorance may distinguish abortions from non-abortive acts. Someone who does not know that her action is likely to cause fetal death, for instance, may be said not to perform an abortion when terminating pregnancy. In 7 of these 19 jurisdictions, language varies between statutes. One Colorado statute, for example, asserts that abortion performed on minors occurs when providers act “with knowledge that the termination by those means will, with reasonable likelihood, cause the death of the minor’s offspring” [31]. Yet, when defining ‘abortion’ generally, a different Colorado statute omits this knowledge requirement [32]. Again, there is nothing inherently wrong with contextualizing different definitions of ‘abortion.’ But it is hardly surprising when doing so causes confusion.^6^

### Knowledge of pregnancy

Another knowledge requirement may separate abortion from non-abortive acts: knowledge that patients are pregnant (see Table 6).

**Table 6:** Knowledge of pregnancy

Explicitly, does performing an abortion require knowledge that the patient is pregnant?		
Yes, the patient is “known to be pregnant.” (*n* = 12)		AK, DE, IL, KY, MN, NC, NJ, NM, RI, TX, UT, WV
No …	… but pregnancy is “clinically diagnosable.” (*n* = 4)	AZ, MA, PA, VT
	… but there is “reason to believe” the patient is pregnant. (*n* = 1)	CO
	… with no further qualifications. (*n* = 17)	CA, CT, FL, HI, IN, KS, MD, ME, MI, MO, NV, NY, OH, OR, SD, VA, WA
Both ‘Yes’ and ‘No’ in some form. (*n* = 14)		AL, AR, GA, IA, ID, LA, MS, MT, NE, NH, OK, SC, TN, WI
‘No’ in multiple forms. (*n* = 2)		ND, WY

The strictest requirement says that an abortion occurs only when the act is performed on someone *known* to be pregnant. In principle, this means someone could avoid performing an abortion if they are uncertain about whether the recipient (including oneself) is pregnant. Women who *suspect* they are pregnant could then self-administer medication designed to terminate pregnancy and, given their uncertainty, this may not qualify as an abortion (strictly speaking).

Perhaps to avoid these kinds of loopholes, 24 states unequivocally lower the bar, stating that pregnancy needs only to be ‘clinically diagnosable’ or that knowledge of pregnancy is irrelevant when distinguishing abortion from non-abortive acts. One also sees 16 states making contextualized (if not conflicting) claims on this matter.

In sum, there is no consensus in U.S. statutory law on ‘abortion.’ There are (at least) six questions to consider when defining ‘abortion’ in law. How should one respond to these inconsistencies? I propose that one answer the six questions in whatever way would best facilitate the development of clear public policy in this (polarized) society. I will endeavor to do so in Sect. Answering the six questions4. Not everyone agrees with this strategy, however. Some authors, like Donley and Kelly, insist that confusion over ‘abortion’ is primarily the fault of anti-abortion legislators who, in effect, have usurped the role of medical experts when defining ‘abortion’ and related terms [10]. Allow me to consider this claim.

## Are legislators the problem?

Perhaps legislators, especially anti-abortion legislators, are to blame for confusion over ‘abortion.’ One might think that the medical community, by comparison, does not suffer from this problem. As one anonymous reviewer remarks, “in medicine, ‘abortion’ as a term is clearly defined. An abortion is the removal or threatened removal of pregnancy tissue, products of conception or the fetus and placenta (afterbirth).” The reviewer adds, “while lawmakers may disagree on definitions in their attempts to limit access to medical and procedural abortions, the medical definition of the term has never varied over decades of practice and training.” So, if anyone is to blame for confusion over abortion, it is *legislators*, not medical experts.

An exhaustive survey of medical literature—to test whether ‘abortion’ is defined consistently throughout—goes beyond the scope of this essay. So does an empirical survey to determine whether ‘abortion’ is defined consistently by providers. It would certainly be interesting to survey a large number of providers regarding the six questions listed above.^7^ But I cannot do that here. Still, I can at least *begin* to test the objector’s claim. If the objector is correct, then there should be relatively homogenous answers to each of the six questions throughout medical literature. At a quick glance, however, such homogeneity is not there.

Consider the following definitions of ‘abortion,’ which come from medical dictionaries, resources for clinical guidance (like UpToDate), and statements by professional organizations, like those made by the American College of Obstetricians and Gynecologists (ACOG). Here is a sample of how ‘abortion’ is defined by these sources:

- **Reviewer’s Definition:** Abortion is “the removal or threatened removal of pregnancy tissue, products of conception or the fetus and placenta (afterbirth).”
- ***Williams Obstetrics*:** Abortion is “the spontaneous or induced termination of pregnancy before fetal viability” [34, p. 198].
- ***Merriam-Webster Medical Dictionary*:** Abortion is “the termination of a pregnancy after, accompanied by, resulting in, or closely followed by the death of the embryo or fetus” [35].
- **ACOG:** Abortion is “an intervention intended to terminate a pregnancy so that it does not result in a live birth” [36].
- **UpToDate:** Abortion “refers to pregnancy loss up to 20 weeks (sometimes called spontaneous abortion) or pregnancy termination at any gestational age” [37].

None of these sources define ‘abortion’ in the same way. The definitions are not merely reworded assertions of the same claim either. They differ on substantial matters. This fact is sometimes overlooked. When praising the medical profession for consistency in defining ‘abortion,’ for example, Donley and Kelly cite the definitions of ‘abortion’ from both *Williams Obstetrics* and the *Merriam-Webster Medical Dictionary*, quoted here [10, 34, 35]. Ironically, Donley and Kelly fail to appreciate that those two sources define ‘abortion’ quite differently. Here, therefore, are three examples of how these definitions differ in substantial ways.

First, on the reviewer’s definition, ‘abortion’ refers to every delivery, since it includes ‘removal’ of ‘the fetus and placenta’ without qualification. There is no specification as to whether delivery results in a live birth or not. Nor does it say whether delivery occurs before viability or after. Removal of the fetus and placenta occurs in *all* cases of birth, however. So, on the reviewer’s definition, all born human beings have been aborted. By contrast, on the definition from *Williams Obstetrics*—which Donley and Kelly hail as “the preeminent Obstetrics textbook” [10]—abortion applies only to events that occur “before fetal viability” [34]. Deliveries that occur after fetal viability are, by definition, non-abortive acts. So, *Williams Obstetrics* defines ‘abortion’ in a substantially different way than the reviewer. The former is more restrictive than the latter, since the reviewer’s definition refers to all ‘removals,’ while the *Williams Obstetrics* definition only refers to some of them.^8^

Second, ACOG explicitly builds intention into its definition of ‘abortion’ [36]. The other four sources do not. This means that what counts as abortion under ACOG’s definition will not always count as abortion under other definitions. To illustrate, for decades, Catholic bioethicists and physicians have argued that removal of an ectopic pregnancy via salpingectomy is not necessarily an abortion—at least not a *direct* abortion—because such removals do not require acting with an intention to cause the death of the embryo (or prevent live birth). Rather, such acts may be performed with the intention to remove a diseased organ (i.e., the tube which will likely burst if left untreated) even though the act will result in the unintended death of the embryo [38, 39].^9^ ACOG’s definition is consistent with these claims. That is, both ACOG and Catholic scholars will, at times, agree that certain interventions do not count as abortions, even if the ethical reasoning behind their respective judgments differs significantly. Specifically, an ectopic pregnancy may be removed with no intention to prevent live birth. So, under ACOG’s definition, removal of an ectopic pregnancy (and removal of other previable pregnancies, for that matter) will not necessarily count as ‘abortion.’ This squares with the perspective of certain Catholic scholars mentioned above. Moreover, the American Association of Pro-Life Obstetricians and Gynecologists (AAPLOG)—which is comprised, in part, by medical experts—makes the same claim, asserting that treating ectopic pregnancy “is not the same thing as performing an abortion” [40]. Yet, removal of an ectopic pregnancy *will* count as an abortion under other medical definitions of ‘abortion.’ After all, such removals will always be ‘closely followed by the death of the embryo.’ So, under the *Merriam-Webster* definition, these removals would likely count as abortions [35].

Which definition is correct? Which is canonical? These are the questions that should be considered, both within the medical community and beyond it. What should not be tolerated are undefended (not to mention incorrect) assertions that the medical community currently possesses clear, consistent, canonical answers. So, when one anonymous reviewer asserts—without defense—that “from a medical perspective, abortion is required to treat ectopic pregnancy is absolutely true,” the above discussion shows that this claim is, at most, *contingently* true. It is contingent upon *which* ‘medical definition’ one adopts. Given ACOG’s definition (and AAPLOG’s statement), the reviewer’s claim is false. Given the *Merriam-Webster* definition, the reviewer’s claim is true. Who is right (and why)? These are conversations worth having.

Third, UpToDate’s definition extends ‘abortion’ to “termination at any gestational age” [37]. In doing so, it contradicts the *Williams Obstetrics* definition, since the latter only concerns acts performed prior to fetal viability. Stating that abortion involves ‘termination at any gestational age’ does not specify whether fetuses survive the process either. So, UpToDate’s definition does not obviously refer to the same phenomena captured by the *Merriam-Webster* definition, which *requires* fetal demise. Thus, a cursory glance suggests that ‘medical definitions’ of ‘abortion’ differ in terms of which gestational age range is relevant, whether intention is relevant, whether fetal demise must occur, and so on.^10^ Bald assertions that there exists widespread consistency on ‘abortion,’ therefore, do not appear to be grounded in fact.

The objector could respond that medical experts all *know* what ‘abortion’ means, even if their formal writing on the topic suggests otherwise. That strikes me as implausible if not intellectually dishonest. If, by and large, medical experts all agree on the meaning of ‘abortion,’ one would expect far more consistency in ‘authoritative’ texts and statements than the evidence reveals. Still, one might think that I am engaged in fruitless nitpicking. In fact, one anonymous reviewer accuses me of “playing definitional games” which “is not only dangerous but also disrespectful.” I disagree. I am seeking and advocating for clarity. Continued ambiguity, inconsistency, and confusion, whether in legal contexts or medical contexts, likely risks lives. Insisting that there is clarity—when, in fact, there is not—only obscures the problem. In my view, *denying* that a real problem exists, especially *without defense*, is far more ‘dangerous’ than revealing the problem and seeking to resolve it.

In sum, my main claim in this section has been that there does not appear to exist a canonical ‘medical definition’ of ‘abortion’ today, let alone a definition that has “never varied over decades” (to borrow an anonymous reviewer’s phrase). The medical domain resembles the legal domain in this regard: ‘abortion’ is highly ambiguous. Lest I risk alienating medical experts further, however, observe that this finding is compatible with thinking that medical experts are *the most important participants* in conversations about how ‘abortion’ should be defined. Outsiders need not encroach upon the medical community’s territory, nor must they unilaterally impose upon the medical community their own definitions. Rather, my view is that there are compelling reasons to seek clarity on ‘abortion’ across legal, medical, and academic domains. Who should ‘lead the charge’ is open for discussion.

No matter who is thought most qualified to define ‘abortion,’ however, there need not be an adversarial relationship between medical and non-medical personnel here. An interdisciplinary approach is perfectly possible. And, given that abortion raises ethical, legal, medical, biological, cultural, and metaphysical questions, an interdisciplinary approach seems necessary anyway. Fortunately, professionals trained in law, medicine, philosophy, etc., can work together towards developing clear language, definitions, and policy. Such an endeavor would truly be in the spirit of bioethics as well, since bioethics is inherently interdisciplinary. Indeed, as professional bioethics came into being, Daniel Callahan observed “an unwritten rule that no one, under any circumstances, should try to pull disciplinary rank” [42, p. 28]. When medical experts (or others) ‘pull disciplinary rank’—by insisting that they alone control some space that clearly calls for interdisciplinary discussions—doing so both impoverishes and runs contrary to bioethical inquiry.

This essay, by contrast, embodies an interdisciplinary spirit. It provides a framework that highlights critical aspects of ‘abortion’ to consider when defining our terms across domains. It does so, moreover, in a way that is neutral with respect to the morality of abortion. As argued above, in pursuit of clarity in public policy, the framework provides six questions to consider when defining ‘abortion.’ I conclude by proposing how one might best answer these six questions and how one might best define ‘abortion’ within a polarized society.

## Answering the six questions

When considering the six questions as they pertain to public policy, there are two options. First, one could allow every citizen to answer each question as they see fit. That is, the state could simply stay out of the matter. Second, one might give well-reasoned answers to each question, which could then inform public policy. The second approach is preferable in a polarized society. Here is why.

On the first option, it may seem that allowing every individual to decide for themselves what ‘abortion’ means exemplifies a commitment to individual liberty and pluralism. As one anonymous reviewer argues, this approach “would be a truly pluralistic response” to resolving disputes over abortion. Appearances aside, however, this approach would entail a surrender of policy to abortion proponents. As Beckwith explains, in the now-defunct *Roe v. Wade*, Justice Blackmun argued that “the word ‘person,’ as used in the Fourteenth Amendment, does not include the unborn” [20, p. 27]. Yet, if the word “person” *did* include the unborn, Blackmun conceded, then under the U.S. Constitution, any right to abortion “collapses,” since the Fourteenth Amendment would protect the “fetus’s right to life” [20, p. 30]. Here, the Supreme Court openly admitted that the right to abortion seemingly *requires* that states deny the personhood of ‘the unborn.’

Suppose, then, that the state allows everyone to define ‘abortion’ and obtain one as they see fit. This policy commits the state to the claim that embryos and fetuses are not persons under the Constitution. Yet, that is *the very claim* that abortion opponents often dispute [20, 43]. This means that the state cannot be neutral when it comes to abortion [20]. Allowing legal access to abortion requires excluding ‘the unborn’ from counting as persons. So, allowing every citizen to define ‘abortion’ as they see fit (and obtain one at will) works, as a policy, only if abortion proponents’ beliefs and values are codified into public policy at the most fundamental level. Thus, this proposal is not a viable solution within a polarized society like the U.S. The ‘solution’ will appeal only to abortion proponents. If allowing every citizen to define ‘abortion’ as they see fit is not a real solution, therefore, I propose that one works to provide well-reasoned answers to each of the six questions, which can then inform public policy.

### Defining ‘pregnancy’

Disputes over the meaning of ‘pregnancy’ are well-documented [44]. Medical experts often state that ‘pregnancy’ begins at implantation. As Rachel Gold notes, claiming “pregnancy begins at conception” would mean going “against the long-standing view of the medical profession” that says pregnancy begins at implantation [13, p. 7]. ACOG states that “fertilization … is the first step … that *leads to* pregnancy” [45, emphasis added]. The NIH and NHS concur [46, 47]. So, pregnancy purportedly begins *after* conception, not at conception. Yet, in ordinary language, ‘pregnancy’ is commonly defined as beginning at conception [48]. Which definition is correct (and why)? Answering this philosophical question is beyond the scope of medical science.

Some readers may disagree. One may think that defining ‘pregnancy’ *is* strictly a medical matter, in that pregnancy begins when human chorionic gonadotrophin (hCG) is produced. Since this occurs sometime after conception, pregnancy cannot be said to begin *at* conception [9]. Yet, does hCG production mark the *start* of pregnancy or is it merely an *effect* of pregnancy already underway? What medical (or scientific) reason is there to favor one answer over the other? Further, when one insists that production of hCG marks the start of pregnancy, I worry that this is equating pregnancy with *detectable* pregnancy. By comparison, there is a difference between having cancer and having cancer that is detectable. Cancer does not begin to exist the moment it becomes detectable. Nor does cancer begin to exist the moment it is detected. The same can be said for pregnancy.^11^ I understand that the medical community may *choose* to define ‘pregnancy’ in a particular way. But that is different from showing that there are good medical or good scientific reasons for doing so. Absent compelling reason for their choice, a collective endorsement of defining ‘pregnancy’ as beginning at implantation looks like an arbitrary pragmatic decision.

Fortunately, when it comes to public policy, one can bypass this debate. The central ethical issue in debates over abortion is the treatment of embryos and fetuses (henceforth, ‘embryos’).^12^ If the abortion debate is fundamentally over the treatment of embryos, then as a matter of public policy, interventions should be sorted clearly into two categories: those that harm embryos and those that do not. If interventions that could potentially harm embryos are clearly labeled as such, then the definition of ‘pregnancy’ is not especially important. As is, given that ‘pregnancy’ is often defined by medical experts as beginning at implantation, this provides room for medical professionals and pharmaceutical companies to obscure the deleterious effects that some interventions may have on pre-implanted embryos [57]. One worry with defining ‘pregnancy’ as beginning at implantation, therefore, is that some interventions that harm embryos might not be clearly marketed as such.

If public policy states that pregnancy begins at implantation, then anti-implantation interventions—which prevent embryos from implanting in the uterus and so, harm them, even if only indirectly—may be labeled ‘contraceptives.’ Imagine, then, that anti-implantation interventions are marketed or prescribed as ‘contraceptives’ on the basis that they prevent implantation and, therefore, prevent ‘pregnancy.’ For women who think that pregnancy begins at conception and who wish not to kill embryos, they may use these interventions thinking they prevent *conception* only. Hence, some women may be misled into unknowingly doing things they regard as morally abhorrent. Defining pregnancy as beginning at conception would eliminate this risk, as it would require that anti-implantation methods be marketed (and prescribed) as abortifacients.

Still, if ‘pregnancy’ is defined as beginning at conception, this could enable restrictive states to ban access to known (or suspected) anti-implantation interventions. This would limit the reproductive freedoms available to women in restrictive states. Additionally, in a world where ‘pregnancy’ is defined as beginning at conception, those who believe that pregnancy begins at implantation might be misled (by their own lights) into believing that anti-implantation interventions are abortive. Someone who is opposed to abortion, but not anti-implantation interventions (since she judges that these are contraceptives) might be misled into refusing an anti-implantation intervention. This, in turn, could lead to her becoming pregnant—or carrying a pregnancy—that she would have prevented, had she been properly informed regarding how a given intervention works. So, there are risks to informed reproductive choice no matter how ‘pregnancy’ is defined.^13^

These risks could be avoided if interventions that prevent (or interrupt) pregnancy are classified, clearly, in terms of their (potential) effects on embryos. Those who object to anti-implantation methods—and who would categorize these interventions as ‘abortifacients’—could then make informed choices about which interventions to use or refuse. Those who do not object to anti-implantation methods would be properly informed as well. Thus, when defining ‘abortion,’ it seems best to avoid taking a stance on the meaning of ‘pregnancy’ and, instead, focus explicitly on what interventions do to embryos. For that reason, my definition will avoid the word ‘pregnancy’ and will focus on interventions that harm embryos in vivo, whether implanted or not.

### Prescribing should not count as performing an abortion

Next, prescribing an abortifacient differs from performing an abortion. By analogy, suppose I provide poison to an assassin with the intent that he uses it to kill his target. I do not thereby assassinate the target. The assassin does. Or, if he changes his mind and *discards* the poison, then nobody is killed (i.e., no assassination occurs). Similarly with abortion, by providing someone with the means to perform an abortion, I do not thereby perform one. Imagine my patient never fills their prescription. If prescribing an abortifacient means performing an abortion, then in this case, I performed an abortion even though no pregnancy is terminated. That is absurd. As such, one should reject definitions of ‘abortion’ that state the act of prescribing an abortifacient is the same as providing an abortion.^14^ Moreover, restrictive states can still maintain that prescribing abortifacients is a serious, punishable crime. In other words, just because the act is not the same as performing an abortion, that does not mean that restrictive states must tolerate the act. Whether one opts for a restrictive or permissive policy, therefore, one’s policy need not—and should not—confuse abortive acts with the enabling of abortive acts.^15^

### Termination (of life) must occur

Abortion is best understood as an act that ends the life of an embryo or fetus. Thus, it is not just an act performed ‘with the intention to terminate pregnancy.’ After all, if abortion were merely an act done ‘with the intention to terminate,’ then there would be no meaningful distinction between abortion and *attempted* abortion. This is counterintuitive.

My proposed answer also clarifies that not *every* termination of pregnancy is an abortion. All vaginal deliveries and all cesarean sections terminate pregnancy, but they are not all abortions [58]. Saying otherwise does not remotely track ordinary language use. Nor would it track the contours of debates over abortion, since abortion opponents are not opposed to termination of pregnancy, per se. Rather, abortion opponents oppose a *kind* of termination—one that involves harming embryos—while abortion proponents claim that such terminations are permissible (and should be legal). In public debate and policy, one’s language should acknowledge these differences. That is, one’s language should reflect the fact that abortion involves the termination of pregnancy *in a certain way*, so that not all terminations are counted as ‘abortions.’^16^

On my proposal, the death of the embryo or fetus is a *necessary* condition for abortion (even though it is not a sufficient condition). This claim is also supported by the fact that, colloquially, there are two kinds of ‘failed’ abortions: one in which pregnancy continues and one in which a fetus is ‘accidentally born alive’ [59, 60]. Since ‘failed abortion’ (or, crudely, ‘botched abortion’) sometimes refers to cases where fetuses are born alive, then plausibly, the death of the embryo or fetus is considered a necessary condition for abortion (in ordinary language). If so, then acting with the intention to end an embryo or fetus’s life—and failing to do so—is an *attempted* abortion at most. A compelling definition of ‘abortion’ will reflect these points.

### Problems for intention-based definitions

As shown in Sect. "Six points of disagreement in U.S. statutory law", when defining ‘abortion’ in terms of intentions, states usually adopt one of two strategies. Some claim that abortion requires acting with a particular intention (e.g., an ‘intention to kill’ an embryo or fetus). Most states, however, claim that abortion involves termination of pregnancy with any intention *other than* an intention to ‘produce a live birth,’ ‘treat an ectopic pregnancy,’ etc. I worry about both approaches.

If abortion requires having a specific intention, then actions to which ‘abortion’ refers seem too limited. Consider someone who fears that continued pregnancy will lead to her unemployment. At 7 weeks’ gestation, she ingests a prescription designed to end pregnancy abruptly. Killing an embryo may never have entered her mind. She may simply act with an intention *not to be pregnant*. If, to count as an abortion, the agent must *intend the death* of the embryo (in some overt, conscious sense), then an abortion does not occur here. Neither abortion proponents nor abortion opponents should accept this implication. Abortion proponents often argue that cases like this—where continued pregnancy threatens one’s job security or financial well-being—make abortion access necessary in the first place [61]. The intervention is also a paradigmatic example of something that abortion opponents oppose. If a definition of ‘abortion’ does not capture this (paradigmatic) case, therefore, it should be rejected.

If abortion involves termination of pregnancy with any intention ‘*other than*’ some specified intention(s), the above problem goes away. On these (‘other than’) approaches, acts of termination are considered ‘abortions’ until proven otherwise. Three problems arise for these approaches.

First, the more one includes on lists of intentions that, if possessed, render an act non-abortive, the murkier abortion policy becomes. This is especially concerning in restrictive states. Above, reports were shared of clinicians who (mistakenly) judge that ectopic pregnancy cannot be treated—since it involves the termination of pregnancy—even though restrictive laws usually state that acting with an intent to treat of ectopic pregnancy does not constitute an abortion [3, 4, 6]. Such subtle distinctions and qualifications are easy to overlook. After all, on these (‘other than’) approaches, clinicians must understand (a) how ‘abortion’ is defined, (b) which intentions render an act a non-abortive, and (c) any exceptions in law which allow for the performance of ‘medically necessary’ abortions.^17^ Clinicians cannot be faulted for confusion here. The more one includes on a list of intentions that render acts ‘non-abortive,’ the harder it is to remember and apply them all.

Second, the more one adds to one’s list, the more ad hoc one’s definition of ‘abortion’ becomes. Principles of parsimony require that one avoid complicating definitions unnecessarily. If, to remain plausible, a definition requires extensive qualifications—as ‘other than’ approaches seem to do—then one wonders if the term is really picking out a genuine category of action.

Third, not only do ‘other than’ approaches complicate policy, but they remain incomplete. To illustrate, suppose I fire a bullet at my friend’s shoulder with the sole intention of preventing a mosquito from biting him. When questioned, I truthfully assert that I intended only to remove the mosquito. Pure intentions will not absolve me of wrongdoing because *I should have known better*. Beyond intentions, therefore, knowledge and circumstances are also relevant when examining one’s actions. This means that, somewhat paradoxically, ‘other than’ approaches are both burdensome and incomplete.

In sum, intention-based definitions of ‘abortion’ face problems. ‘Other than’ approaches are difficult to implement. They add complexity, perhaps in an ad hoc way, and invite confusion. They also fail to distinguish abortions from non-abortive acts adequately. Approaches that say abortion requires having some specific intention(s), however, risk making the referent of ‘abortion’ unduly narrow. If a third option exists—one that avoids mention of ‘intention’—that may be best. So, my proposed definition of ‘abortion’ will avoid any explicit mention of ‘intention.’

### Knowledge of death is unnecessary

Performing an abortion should not be thought to require knowledge that one will (likely) cause the death of an embryo. Such knowledge is more relevant to questions about responsibility. To illustrate, consider three cases. First, a pregnant woman ingests an abortifacient, knowing that it will likely cause the death of her embryo. Second, a pregnant woman ingests an abortifacient handed to her, not knowing what it is or what it will do. Third, someone slips an abortifacient into a pregnant woman’s drink without her knowledge. Embryos die in all three cases. Does an abortion occur in all three cases? Plausibly so.

The first case describes a paradigmatic example of abortion. In the second case, an abortion occurs even though the agent did not ask any questions about what she was ingesting. Abortion opponents can argue ‘she should have known better’ than to ingest an unknown substance and so, restrictive states may find her *responsible* for having obtained an abortion. But her lack of knowledge does not imply that an abortion did not occur. In the third case, an abortion occurs, but given the agent’s lack of knowledge, she is *not* responsible for it. Instead, she looks like a victim of *coerced* abortion [62]. If so, then agents’ knowledge of potential harm is not what separates abortions from non-abortive acts, but agents’ knowledge helps determine responsibility (or legal culpability) for the action performed.^18^

In restrictive states, people who perform abortions can be held *responsible* for doing so when there is a relevant knowledge requirement and that knowledge requirement is met. Agents may be deemed ‘not responsible’ when the knowledge requirement is present but not met. Whether one is responsible for an abortion, however, is a separate question from whether an abortion occurs. So, factoring into definitions of ‘abortion’ an agent’s knowledge (or what they *should* know) seems like a mistake, even if assessing agents’ knowledge is useful for other purposes.

### Knowledge of pregnancy is unnecessary

Finally, performing an abortion should not be said to require knowledge of pregnancy. If pregnancy must be known for an abortion to occur, then it is too easy to avoid performing an abortion. With self-administered medication, for example, someone who believes (but does not *know*) that she is pregnant could ingest an abortifacient and subsequently claim not to have performed an abortion. Definitions with such large loopholes do not warrant widespread support.

Neither should ‘abortion’ be restricted only to cases where pregnancy is ‘clinically diagnosable.’ This is because pregnancy is diagnosable around two weeks after conception [12]. If, by definition, actions performed prior to pregnancies being clinically diagnosable are never abortions, then any intervention that kills embryos during the first two weeks is not an abortion. The crux of the abortion debate is the treatment of embryos, however, so this definition of ‘abortion’ will not do. To better track public debates, interventions that harm embryos should be distinguished from those that do not. After all, interventions that kill embryos are the very thing that are in dispute, whether embryos are detectable, implanted, or not. So, a definition of ‘abortion’ should not apply only to ‘clinically diagnosable’ pregnancies.

This leaves two options. Either abortion applies only when women have ‘reason to believe’ they are pregnant, *or* knowledge of pregnancy is irrelevant to ‘abortion.’ Problems arise for the former option. For many women, being sexually active may provide *some* reason to believe that they are pregnant. If so, then under restrictive laws, many women may be unable to obtain medication (or other procedures) that threaten to harm embryos, regardless of how sure or unsure they are about being pregnant. Abortion proponents will not accept this result. Practically speaking, they have reason to claim that abortion occurs only when pregnancy is *known*. That would promote access to the widest range of interventions for women who do not know whether they are pregnant. But again, one should avoid building such major loopholes into public policy.^19^

It looks like problems follow if ‘abortion’ refers only to cases where pregnancy is known, clinically diagnosable, or suspected. No such problems follow if definitions of ‘abortion’ make knowledge of pregnancy irrelevant. So, my definition of ‘abortion’ will not refer to one’s knowledge of pregnancy. On my view, one may still perform (or obtain) an abortion even if pregnancy is not known, detected, detectable, or suspected.

## A proposed definition of ‘abortion’

No matter how one defines ‘abortion,’ one’s definition should align with the best answers to each of the six questions considered above. What the best answers are is up for debate. I have provided some suggestions. Specifically, when defining ‘abortion,’ one should: avoid using the word ‘pregnancy,’ avoid implying that prescribing abortifacients is equivalent to performing an abortion, specify that termination actually occurs, avoid language of ‘intention’ (if possible), and avoid requiring that providers know either that their acts will (likely) harm embryos or that their patients are pregnant. To effectively track public debates, one should also ensure that one’s definition of ‘abortion’ captures all and only types of acts that are in dispute (insofar as is possible).

Here is one definition that comes close to accomplishing all of this. Where ‘*e*’ refers to a zygote, embryo, or fetus in vivo:

**Abortion =** An act that, via medicinal or surgical intervention, secures the death of *e* without regard for *e*’s survival.

In closing, I will unpack this definition, consider one major objection to it, and outline several ways in which it may facilitate development of clear policy.

### Unpacking the definition

First, abortion is an act. It is not something that occurs naturally or randomly but is something that is done.^20^ Moreover, abortion is a specific *kind* of act: it involves either some ‘medicinal intervention’ or ‘surgical intervention.’ The former includes medications like methotrexate, homeopathic methods, etc. The latter includes things like dilation and curettage (D&C), dilation and evacuation (D&E), and so on. By specifying that abortion involves either a medicinal or surgical intervention, it follows that not *every* act of securing an embryo’s death counts as an abortion. This part of the definition serves as a corrective to those who define ‘abortion’ simply as ‘feticide,’ the ‘the intentional or unjust taking of pre-natal human life,’ or ‘the wrongful killing’ of an embryo or fetus, as anti-abortion philosophers often do [64–66].

To see why, imagine that a terrorist bombs a hospital, with an intent to kill everyone inside (including several pregnant women and their fetuses). Suppose that, regrettably, the terrorist succeeds. This event includes several acts of intentional feticide. But it seems wrong to say that the terrorist ‘performed an abortion’ on the fetuses he killed. If correct, then there are cases of intentional feticide (or ‘wrongful killing’ of fetuses) that do not count as abortions. My proposed definition respects this distinction. Accounts that define ‘abortion’ as things like ‘feticide’ or ‘intentional feticide’ do not [64–66].

Relatedly, the definition does not invoke the ‘killing/letting die’ distinction (e.g., by defining ‘abortion’ as a form of killing). There are two reasons for this. First, scholarly debates over the killing/letting die distinction are complicated [67]. If it is possible to avoid getting into the weeds of such discussions—and if one can remain neutral on the nature of the distinction—then so much the better. After all, if one can remain neutral on the distinction, then one need not take on board contentious metaphysical commitments when advancing or applying the definition. Second, by saying that abortion ‘secures the death of *e*,’ it remains possible that death may be secured via an act of killing or via an act of ‘letting die.’ This allows for the possibility that “disconnect abortions” are genuine abortions, where one may “‘merely’ disconnect the fetus, letting it die.” [68, p. 562] In other words, performance of a hysterotomy or hysterectomy may still count as an abortion in some cases, even supposing that a fetus is not killed directly.^21^The definition also avoids the word ‘pregnancy’ and instead, focuses on acts that are harmful to embryos or fetuses (henceforth, ‘embryos’) in vivo. It thereby bypasses debates over when pregnancy begins and maintains a focus on the crux of the abortion debate: how embryos are treated. The definition also implies that merely prescribing an abortifacient does not count as performing an abortion. Prescribing a drug does not secure the death of *e*, even if prescriptions *enable* someone to secure the death of *e*. Thus, one can distinguish between abortion and the enabling of abortion. And, by stating that the act ‘secures the death of *e*,’ the definition is unambiguous: abortion results in fetal demise. Should *e* survive an intervention, therefore, the act is an abortion *attempt* (at most).

The final portion of the definition specifies that abortion involves acting ‘without regard for *e*’s survival.’ This is the closest I come to building intention into my definition. Yet, the definition is not as narrow as those that require a specific intention be present (as in definitions that say abortion only occurs when one ‘intends the death of the fetus’). Nor is the definition an ‘other than’ approach, since it does not list intentions that would preclude one’s action from counting as an abortion. So, I avoid advancing either of the two kinds of intention-based definitions discussed above and, therefore, avoid problems facing both.

Still, intention is relevant to the definition indirectly. When saying that abortion is an act performed ‘without regard for *e*’s survival,’ this *includes* acts of feticide that are performed with the intent, purpose, or desire to secure *e*’s death. By intentionally killing *e*, one shows a lack of regard for *e*’s survival. But the definition goes beyond those cases and includes some acts of feticide that are accompanied by *indifference* to *e*’s survival. This means that abortion may occur even if one is not consciously intending (or desiring) the death of *e*.

One may worry that it seems difficult to establish whether someone acts ‘without regard for *e*’s survival.’ When it comes to establishing this fact, however, precedent in law is readily available. Involuntary manslaughter, for example, is a form of “homicide that results from a reckless or negligent act that the actor commenced without any intent to cause death” [69]. This category of action sets intention aside—as my definition does—and considers whether the agent acts in a ‘reckless’ way. “Recklessness,” moreover, occurs when a person creates “significant risk of harm to others” and is “either aware of the risk and makes no effort to abate it or who is indifferent to the risk” [70]. These are standards already available, therefore, to help establish when someone acts without regard for the life of another being.^22^ This is *not* to say that people who obtain abortions act recklessly or commit manslaughter. Rather, the point is that there are clear, conceptual precedents in law that can help to establish whether someone acts ‘without regard’ for another being’s survival. My definition does not make matters unduly opaque, therefore, since it is possible to determine whether someone acts ‘without regard’ for *e*’s survival.

Finally, acts that directly and intentionally kill embryos—which I consider to be paradigmatic instances of abortion—will count as abortions on this definition. Many interventions directly disrupt *e*’s bodily integrity in lethal ways, as in cases of suction, dilation and curettage (D&C), dilation and evacuation (D&E), lethal injections of potassium chloride into the heart, ‘partial-birth’ abortion, and so forth. It would be absurd for a provider to claim that they are acting ‘with regard’ for *e*’s survival by fatally disabling *e*, dismembering *e*, or injecting *e* with a lethal substance. So, employing those interventions will count as performing an abortion (assuming that *e* dies, that is). This means that—in addition to aligning with my suggested answers to the six questions—the proposed definition also refers to (many) paradigmatic cases of ‘abortion.’ Still, one may worry that the definition does not capture *every* paradigmatic case, as we shall now see.

### An objection: previable hysterotomies and hysterectomies

Here is a potential problem for my definition. Consider two ways of ending previable pregnancies: via hysterotomy and via hysterectomy. For simplicity, let them be understood as follows:

**Hysterotomy:** A previable fetus is developing in utero, the uterus is surgically opened, and the fetus is removed from the mother’s body, fully intact. The fetus dies [71].

**Hysterectomy:** A previable fetus is developing in utero and the entire uterus (including the fetus) is removed, fully intact. The fetus dies [72].

One might argue that whether a hysterectomy or hysterotomy is performed prior to viability, both should count as an abortion. If either procedure is implied by my definition *not* to be an abortion, then that is a problem.^23^

In response, my definition implies that some hysterotomies and hysterectomies will count as abortions, but not all of them will. Hysterotomies and hysterectomies performed *without regard for e’s survival* will count. Someone who obtains a hysterectomy, for example, with the intent that *e* dies would be seeking an abortion. The same applies to someone who opts for a hysterotomy following an adverse prenatal diagnosis. For example, suppose that at 10 weeks’ gestation, a fetus is found to have a condition that will likely lead to their death during the first few years of their life. Subsequently, the mother obtains a hysterotomy or induced delivery at that stage. This act would count as an abortion under my definition because the act is performed without regard for *e*’s *survival*. Of course, one may argue that the act is permissible, merciful, or performed for the fetus’s benefit, insofar as it spares the fetus and family some degree of suffering. But questions of what is permissible, merciful, or beneficial remain separate from the question of whether an act counts as an abortion.

In contrast to these cases, suppose that a patient with advanced uterine cancer, who desperately wishes that her 12-week-old fetus survives, must undergo a hysterotomy to save her own life. Her fetus has no chance at survival at 12 weeks’ gestation. Still, it is possible for the patient and provider to act with real regard for *e*’s survival. The patient might truthfully state that were there *any* way to save *e*, she would pursue it. This would be compelling evidence that she is, indeed, sufficiently concerned for *e*’s survival. Under those circumstances, even though this is a form of previable delivery, my definition would not label it an ‘abortion’ (at least not obviously).^24^

This claim may seem absurd. Surely a hysterotomy, when performed at 12 weeks’ gestation, is either an abortion in all cases or an abortion in none of them. I am not convinced. By comparison, consider a case in which one surgeon severs their patient’s artery as part of routine heart transplant. Meanwhile, another surgeon severs their own patient’s artery—the same artery, with the same type of blade, at the same rate, etc.—and lets the patient bleed to death. Did both surgeons perform *the same action*? Yes and no. Mechanically, both did the same thing. They both applied a blade to an artery and severed it. Stopping there would leave us with an impoverished description of their respective actions, however. That is because the mechanics and tools involved do not tell the whole story. One surgeon engaged in an act of killing, the other did not. A *complete* description of the surgeons’ respective actions, therefore, will explain why the actions are importantly different.

My definition of ‘abortion’ does not focus exclusively on the mechanics of an action, nor the tools involved. Hence, some previable hysterotomies (or hysterectomies) may count as abortions, while others may not, even if the same *mechanical* process occurs across cases.^25^ By moving beyond mechanics and tools, my definition is pragmatically useful in ways that other definitions—especially the ‘medical’ definitions listed in Sect. 3—are not. My definition is designed to track the contours of the abortion debate. Above, I argued that when defining ‘abortion’ for the purpose of public policy (in a polarized society), the definition should refer to *all and only* the kinds of acts that are in dispute. Abortion opponents do not typically object to various forms of previable delivery when the mother’s life is in jeopardy (at least when *e* is not directly and intentionally killed). [73, p. 233]. So, they generally will not oppose a hysterotomy in the case described above. If those kinds of cases are not captured by ‘abortion,’ therefore, then relevant interventions may be performed without providers running afoul of abortion restrictions.^26^

To add some further subtlety, a mother who opts for a hysterotomy (or hysterectomy) may reasonably be said to have sufficient regard for *e*’s survival if the reason she seeks the procedure is to save her life. A hysterotomy, in such a case, likely would not count as an abortion. By contrast, someone who opts for a hysterotomy simply because she wishes not to be pregnant—or, to use Thomson’s example, because she wants to go on vacation [74, pp. 65–66]—exhibits a *lack* of regard for *e*’s survival. A hysterotomy in that case would count as an abortion. Thus, my definition counts some previable deliveries as ‘abortions,’ while others do not count. Given my aim—to provide a simple definition that refers to all and only disputed cases, insofar as is possible—this is an advantage of the definition, not a disadvantage.

Lastly, one may ask: where is the line when determining that someone acts ‘without regard’ for the life of another? Abortion opponents will likely set a high bar, using other cases as a guide. They may ask: under what circumstances might a parent justifiably allow their *born* child to die? Perhaps only to save another life (including the parent’s own life) or when their child is already dying. A parent would *fail* to show (sufficient) regard for the life of his born child, however, if he allowed his child to die so that he might go on vacation, avoid financial hardship, keep his job, save his marriage, and so forth. Hysterotomies or hysterectomies performed on previable fetuses for *those* sorts of reasons, therefore, seemingly involve acting without regard for *e*’s survival (and so, would count as ‘abortions’ and would be subject to abortion restrictions). Interventions performed to save the mother’s life, however, would not obviously suggest a lack of regard for *e*’s survival and so, need not count as ‘abortions’ under my definition. Hysterotomies and hysterectomies performed to save the mother’s life, therefore, would not be subject to abortion restrictions, even when performed prior to fetal viability.

One may continue to wonder: how can someone act ‘with regard for *e*’s survival’ when ending pregnancy prior to viability via hysterectomy, hysterotomy, or induced delivery? It may seem that if one *knows* that their action will indirectly result in *e*’s death, then in principle, the act *cannot* be performed with any real regard for *e*’s survival. In response, I have argued that the reasons one has for ending a previable pregnancy shape the nature of the action itself. Opting for previable delivery so that one can go on vacation illustrates a lack of concern—a lack of regard—for *e*’s survival. A person who adequately cares about *e*’s survival will not act in this way. So, under those circumstances, if one were really concerned about *e*’s survival, then one would not opt for delivery at that stage. By contrast, if continued pregnancy were highly likely to result in one’s own death, then previable delivery may be performed *without implying* that one lacks regard for *e*’s survival. An analogy will be helpful here.

Suppose that my neighbor is trapped in a burning building, and I recognize that any attempt to save them will very likely result in my death. It seems to me that, morally and legally speaking, I have no obligation to attempt to rescue them. My refusal to attempt a rescue, moreover, does not imply that I have any disregard for their survival. Rather, ensuring my own survival is a strong enough reason to justify my refusal to attempt a rescue. By contrast, suppose that I see a toddler drowning and refuse to rescue them, simply because I do not want to dampen my shoes. Such an action *would* demonstrate an inadequate regard for the toddler’s survival. Someone who cares about the toddler’s survival would not act in this way.

The key point is that allowing a subject to die does not, by itself, imply a lack of regard for the subject’s survival. It will have this implication in some cases, but not all cases.^27^ Similarly, recall that not every act of killing or letting die counts as manslaughter, legally speaking. Rather, causing death, whether directly or indirectly, may be regarded as manslaughter only when done in a “reckless or negligent” way [69]. So, on my proposal, when a hysterectomy, hysterotomy, or induced delivery is performed prior to viability, the action *may* count as an abortion, but will not always count as an abortion.^28^ The difference is whether one acts with regard for *e*’s survival, where adequate regard for *e*’s survival means having a willingness to secure *e*’s death only under the most dire of circumstances.^29^

### Putting the definition to work

Finally, here are some ways in which the proposed definition provides a basis for clarity. ‘Abortion,’ so defined, does not apply to removal of fetal remains under any circumstance. Suppose, for example, that a D&C is used to remove fetal remains. A D&C may be *mechanically* the same whether it is performed on a living fetus or on a dead fetus. The tools, motions, etc., may be comparable. On my definition of ‘abortion’ though, a D&C performed on fetal remains *cannot* be considered an abortion, since one cannot ‘secure the death of *e*’ if *e* is already dead. Thus, providers may rest assured that when they remove fetal remains—by *any* method—they would not run afoul of abortion restrictions.

Treatment (e.g., chemotherapy) of the mother for some disease or illness need not count as an abortion either, especially if treatment is required to save the mother’s life.^30^ Pursuing such treatment does not imply that one lacks sufficient regard for *e*’s survival. To be clear, under consideration are interventions that do not involve any direct, intentional, or purposeful destruction of *e* (since those acts *would* demonstrate a lack of regard for *e*’s survival). Rather, treating the mother for some life-threatening condition—where treatment may have a negative side effect on *e*—is more akin to hysterotomies (or hysterectomies) performed to save the mother’s life (discussed above). So, on the proposed definition, abortion restrictions would not preclude the use of treatments, like chemotherapy, that aim to save the mother’s life even though they might also (inadvertently) cause fetal death.

Regarding treatments for ectopic pregnancy, the discussion will resemble what was said about hysterotomies and hysterectomies. Some interventions will count as abortions, while others may not. Specifically, there are two classes of interventions to consider: medication interventions and surgical interventions. Regarding medication interventions, different medications work differently. If Cara Buskmiller is correct, then mifepristone merely detaches embryos from their mothers’ bodies [26]. If so, then mifepristone need not count as performing an abortion in some cases. To determine that, one would need to consider whether the agent possesses sufficient regard for *e*’s survival to preclude the act from counting as an abortion. If one is using the drug—and allowing *e* to die—for most reasons (e.g., financial security, lack of social support, etc.), the act likely displays a lack of regard for *e*’s survival. So, mifepristone use is likely to count as abortive in most cases.

Alternatively, Hershenov argues that mifepristone *does* directly harm embryos, rather than merely detaching them (and allowing them to die) [77]. If so, then using mifepristone would count as performing an abortion. Again, it seems difficult to maintain that one acts with regard for *e*’s survival while one is actively and directly destroying *e*. So, determining whether mifepristone is abortive requires, to some extent, understanding how it works. This is not a shortcoming of the proposed definition though. The definition will tell what is (or is not) an abortion after establishing the facts regarding how particular interventions work. If Buskmiller is right, then the definition will not automatically label mifepristone use as ‘abortive,’ though many cases of its use will be abortive. If Hershenov is right, then the definition will label mifepristone use as abortive. Either way, the definition will provide a clear verdict once the empirical dispute is settled. Other medications are less equivocal than mifepristone. Methotrexate has deadly effects on embryos; it secures the death of embryo by acting on them directly [26, 77]. If actively ending *e*’s life is incompatible with possessing sufficient regard for *e*’s survival, then methotrexate use will be defined as ‘abortive,’ even when applied to ectopic pregnancies.

Regarding surgical interventions for ectopic pregnancy (e.g., salpingostomy or salpingectomy), the proposed definition does not necessarily apply to these treatments. What matters is one’s regard (or lack thereof) for *e*’s survival. If a salpingostomy or salpingectomy is performed to save the mother’s life, then—as I have argued—this seems compatible with possessing sufficient regard for *e*’s survival. Thus, abortion restrictions would not prevent access to surgical treatment of ectopic pregnancy. And, if Miller is correct, then since medication is generally not an appropriate treatment for ectopic pregnancy anyway, this means that the best treatment options remain fully (and clearly) accessible under the proposed definition, even where abortion is banned outright [63].

## Conclusion

In this essay, I have identified six major questions that must be answered when defining ‘abortion.’ As is, there is no consistent, canonical definition of ‘abortion’ in law nor in medicine. For this reason, I have argued that legal, medical, and academic domains would benefit from a rigorous discussion of these six questions and the meaning of ‘abortion.’ I have also suggested answers to each of the six questions that seem most reasonable for the purpose of crafting public policy in a polarized society. I ended by proposing a definition of ‘abortion’ that squares with these suggestions and tracks the contours of public debates over abortion.

Whether my suggestions and definition are accepted is (much) less important than the framework created here. By identifying the major ways in which there is disagreement over the meaning of ‘abortion’ (and related terms) one can begin to clarify public debate, policy, and clinical practice. Failure to clarify these matters means that confusion over abortion may persist. Confusion risks the health and lives of many pregnant women. Whether one is an abortion proponent or opponent, this is unacceptable. Parties on both sides have compelling reason to provide clarity on abortion and so, compelling reason to provide answers to the questions that I have raised.^31^

## Appendix

The following statutes were referenced when generating the tables in this article.

- Alabama Code 26-21-2. 2024.
- Alabama Code 26-22-2. 2024.
- Alabama Code 26-23B-3. 2024.
- Alabama Code 26-23D-1. 2024.
- Alabama Code 26-23E-3. 2024.
- Alabama Code 26-23I-2. 2024.
- Alaska Stat. 18.16.090. 2024.
- Alaska Stat. 18.50.950. 2024.
- Arizona Stat. 36-2151. 2024.
- Arizona Stat. 36-2155. 2024.
- Arkansas Code 20-9-302. 2024.
- Arkansas Code 20-16-603. 2024.
- Arkansas Code 20-16-1102. 2024.
- Arkansas Code 20-16-1302. 2024.
- Arkansas Code 20-16-1503. 2024.
- Arkansas Code 20-16-1601. 2024.
- Arkansas Code 20-16-1802. 2024.
- Arkansas Code 23-79-156. 2024.
- California Health & Safety Sec. 123464. 2024.
- California Ins. Sec. 10123.1961. 2024.
- Colorado Rev. Stat. 13-22-703. 2024.
- Colorado Rev. Stat. 25-6-402. 2024.
- Connecticut Gen. Stat. 19a-912. 2024.
- Delaware Code Tit. 16 Sec. 3101. 2024.
- Delaware Code Tit. 24 Sec. 1782. 2024.
- Florida Stat. Ch. 390.011. 2024.
- Georgia Code 16-12-141. 2024.
- Georgia Code 31-9A-2. 2024.
- Hawaii Rev. Stat. 453-16. 2024.
- Idaho Code 18-502. 2023.
- Idaho Code 18-604. 2023.
- Idaho Code 18-8702. 2023.
- Idaho Code 39-9503. 2023.
- Illinois, 775 ILCS 55/1-10. 2024.
- Indiana Code 16-18-2-1. 2024.
- Iowa Code Sec. 146B.1. 2023.
- Iowa Code Sec. 146E.1. 2023.
- Iowa Code Sec. 217.41B. 2023.
- Kansas Stat. 65-6701. 2024.
- Kentucky Rev. Stat. 311.720. 2024.
- Kentucky Rev. Stat. 311.732. 2024.
- Kentucky Rev. Stat. 311.772. 2024.
- Kentucky Rev. Stat. 311.7701. 2024.
- Louisiana Sec. 14:87.1. 2024.
- Louisiana Sec. 40:1061.1.2. 2024.
- Louisiana Sec. 40:2175.3. 2024.
- Maine Rev. Stat. Tit. 22 Sec. 1596. 2024.
- Massachusetts Gen. Laws Ch. 112, Sec. 12K. 2024.
- Michigan Comp. Laws 333.2690. 2024.
- Michigan Comp. Laws 333.17015. 2024.
- Michigan Comp. Laws 333.17516. 2024.
- Michigan Comp. Laws 400.109e. 2024.
- Minnesota Stat. 144.343. 2024.
- Minnesota Stat. 145.411. 2024.
- Mississippi Code 41-41-45. 2024.
- Mississippi Code 41-41-105. 2024.
- Mississippi Code 41-41-153. 2024.
- Mississippi Code 41-41-191. 2024.
- Missouri Rev. Stat. 188.015. 2024.
- Montana Code 50-20-104. 2024.
- Montana Code 50-20-303. 2024.
- Montana Code 50-20-703. 2024.
- Montana Code 50-20-1002. 2024.
- Nebraska Rev. Stat. 28-326. 2024.
- Nebraska Rev. Stat. 28-3,103. 2024.
- Nebraska Rev. Stat. 71-6901. 2024.
- Nebraska Rev. Stat. 71-6914. 2024.
- Nevada Rev. Stat. 442.240. 2024.
- New Hampshire Stat. 132.32. 2024.
- New Hampshire Stat. 329:43. 2024.
- New Jersey SA 9:17A-1.3. 2023.
- New Mexico Stat. 24-14-2. 2024.
- New Mexico Stat. 30-5A-2. 2024.
- North Carolina Stat. 90-21.81. 2024.
- North Dakota Cent. Code 12.1-17-14. 2024.
- North Dakota Cent. Code 12.1-17.1-01. 2024.
- North Dakota Cent. Code 14-02.1-02. 2024.
- Ohio Rev. Code 2919.11. 2023.
- Ohio Rev. Code 2919.16. 2023.
- Oklahoma Stat. Tit. 63, Sec. 1-730. 2024.
- Oklahoma Stat. Tit. 63, Sec. 1-745.51. 2024.
- Oklahoma Stat. Tit. 63, Sec. 1-756. 2024.
- Oklahoma Stat. Tit. 63, Sec. 1-757.2. 2024.
- Oregon Stat. 432.005. 2024.
- Pennsylvania Cons. Stat. 3203. 2023.
- Rhode Island Gen. Laws 23-4.7-1. 2024.
- South Carolina Code 44-41-10. 2024.
- South Carolina Code 44-41-430. 2024.
- South Dakota Codified Laws 34-23A-1. 2024.
- South Dakota Codified Laws 34-23A-45. 2024.
- South Dakota Codified Laws 36-4-48. 2024.
- Tennessee Code 37-10-302. 2024.
- Tennessee Code 39-15-213. 2024.
- Tennessee Code 63-6-1102. 2024.
- Texas Health and Safety Code Sec. 171.201. 2023.
- Texas Health and Safety Code Sec. 245.002. 2023.
- Utah Code 76-7-301. 2024.
- Vermont Stat. Tit. 8 Sec. 4099e. 2024.
- Virginia Code Sec. 16.1-241. 2024.
- Washington Rev. Code 9.02.170. 2024.
- Washington Rev. Code 19.373.010. 2024.
- West Virginia Code 16-2F-2. 2024.
- West Virginia Code 16-2R-2. 2024.
- Wisconsin Stat. Sec. 20.927. 2024.
- Wisconsin Stat. Sec. 253.10. 2024.
- Wyoming Stat. 35-6-121. 2024.
- Wyoming Stat. 35-6-122. 2024.

Insufficient information available for the following jurisdictions: MD, NY.

## Footnotes

1 One may argue that ambiguity and disagreement on ‘abortion’ is isolated to the legal domain. Perhaps the medical community, by contrast, possesses clear, consistent definitions of ‘abortion’ and so, were legislators to follow medical experts on the meaning of terms, all manner of confusion and ambiguity would be eliminated. In the third section, I argue that this is false. ‘Abortion’ is highly ambiguous throughout the medical literature too.

2 To my knowledge, only Donley and Kelly have produced anything like my overview of state definitions [10]. Their work was produced concurrently with (and independently from) my own. That said, they do not provide the same six-variable framework that I develop. Nor do they describe each state’s definition in the same fine-grained detail. And, as will be shown below, Donley and Kelly argue that the solution to confusion over ‘abortion’ in law is to leave medical providers alone and to stop trying to regulate abortion [10, p. 73]. This suggestion will appeal only to abortion proponents, so it does nothing to advance debates over abortion. More importantly, in the third section of this essay, I tackle this suggestion directly, arguing that inconsistent definitions of ‘abortion’ pervade the medical domain too, a fact that Donley and Kelly underappreciate. Thus, contra Donley and Kelly, it is a mistake to look to today’s medical community for clarity on ‘abortion,’ since clarity is elusive there as well.

3 See the Appendix for a list of state statutes referenced when generating the tables in this essay. All tables were generated as of February 14, 2024, so some state statutes may have changed since that time.

4 Collapsing the distinction between performing and enabling also renders unclear the matter of who is complicit (or cooperating with) an abortion when the means of prescribing and taking an abortifacient fall outside of any traditional physician–patient relationship. I am grateful to Cody Feikles for raising this point.

5 Table 4 lets each state answer the fourth question more than once. This is because some states have two or more statutes that answer question 4 differently. Iowa, for example, has one statute that says abortion involves termination with any intention ‘other than’ the intention to remove fetal remains (without further qualification) [27]. A different Iowa statute, however, gives a different answer, stating that abortion involves termination with any intention ‘other than’ the intention to remove fetal remains where fetal death occurred naturally (i.e., in cases of ‘spontaneous abortion’) [28]. So, in Iowa, removal of fetal remains following *induced fetal demise* may, in principle, count as an abortion under the latter statute, but not under the former statute. So, not only are there inconsistencies between states regarding how they answer the fourth question, but some states also possess multiple statutes that answer the same question differently. The source of confusion, therefore, is not limited to interstate inconsistencies. Confusion may also stem from *intra*state inconsistencies, where some states have multiple, conflicting definitions of ‘abortion.’.

6 This example is of interest given that Guttmacher categorizes Colorado as a “protective” state with respect to abortion access [33]. Yet, there are intrastate inconsistencies—which are one source of confusion—within Colorado’s statutes. This renders dubious any suggestion that restrictive states—or anti-abortion legislators—are alone when defining ‘abortion’ in inconsistent or confusing ways, as Donley and Kelly suggest [10].

7 It would be interesting to survey laypeople on those six questions as well. As noted above, doing so might reveal that public polls on abortion are far less meaningful than is sometimes supposed.

8 The definition given by *Williams Obstetrics* is a nonstarter, at any rate, because it implies that abortion cannot occur after fetal viability. This is obviously false. I need not assess the merits of definitions here, however, as my goal is simply to provide evidence that ‘medical’ definitions of ‘abortion’ vary substantially.

9 There is an ambiguity in discussions of ectopic pregnancy within Catholic circles. Some regard treatment for ectopic pregnancy not to be an abortion *at all* (at least when intent to kill an embryo is absent) [38]. Others distinguish between ‘direct abortion’ and ‘indirect abortion,’ where the former involves an intent to kill, but the latter does not (and only the former is categorically prohibited) [39]. Thus, certain treatments for ectopic pregnancy (e.g., salpingectomy, which removes the fallopian tube containing an embryo without acting directly on the embryo) are either non-abortive or *indirectly* abortive (which can be morally justified under certain circumstances). The question to consider, therefore, is whether ‘indirect abortions’ are abortions at all. I take no stance on the matter, but note the ambiguity.

10 As another point of disagreement, some definitions explicitly extend ‘abortion’ to include naturally occurring fetal demise (as in cases of ‘spontaneous abortion’) whereas others do not. The NHS, for example, asserts that “an abortion is different from a miscarriage, which is the loss of your baby before 24 weeks, without medical intervention” [41]. This obviously conflicts with definitions of ‘abortion’ given by *Williams Obstetrics* and *UpToDate*. It also raises the question of whether ‘spontaneous abortion’ should be called ‘abortion’ at all. I am not interested in that question here, however, since my goal is simply to provide evidence that there is no uncontroversial, clear, canonical definition of ‘abortion’ on offer within the medical community.

11 One reviewer pushes back, arguing that “pregnancy is clinically defined by the existence of a positive pregnancy test.” Since a positive pregnancy test cannot exist *at* conception, this implies that pregnancy begins after conception. The existence of a positive pregnancy test does not cause pregnancy, however. A positive pregnancy test may just as well be regarded as *evidence of* a pregnancy that is already underway. Nor is a positive test a necessary condition for pregnancy. After all, if a positive test were a necessary condition for pregnancy, then there could be someone with a 40-week-old, healthy fetus in her uterus who is, by the reviewer’s definition, not pregnant (at least not ‘clinically’). This strikes me as absurd. Worse, some pregnancy tests yield false positives. As such, a ‘positive pregnancy test’ may exist, while a pregnancy does not. The reviewer’s claim implies that a pregnancy *does* exist when a positive pregnancy test exists. So, on the reviewer’s view, it seems there can be no such thing as a false positive. That is obviously false. Regardless, there seems to be a collapse of the distinction between pregnancy, on one hand, and detected (or detectable) pregnancy on the other. These points are mostly tangential, however, so I shall set them aside.

12 Some scholars disagree, claiming that abortion restrictions are generally in place to “control women” [49]. Yet, anti-abortion scholars exhibit significant uniformity in opposing the killing of embryos, not women’s freedoms [50, 51]. The same goes for prominent secular and feminist organizations that oppose abortion: *killing* is the issue, ‘controlling women’ is not [52, 53]. Indeed, many female authors have argued that abortion *itself* is anti-feminist, an expression of sexism, and a ‘masculine’ solution to the ‘problem of unwanted pregnancy.’ [54–56]. Dismissing abortion opposition as inherently ‘sexist,’ ‘anti-feminist,’ or as primarily about ‘controlling women,’ therefore, is unwarranted. This is not to say that ‘controlling women’ is irrelevant to every anti-abortion expression, defender, or movement. Rather, my claim is that it is unreasonable to cast all abortion opposition in this same negative light.

13 In my view, misleading someone into fatally harming an embryo seems morally worse than misleading someone into carrying a pregnancy to term. This is because wrongful killing strikes me as among the worst things that someone can do. So, on balance and with respect to public policy, there may be greater reason to favor defining ‘pregnancy’ as beginning at conception than at implantation. I will not defend this intuition here, however, and certainly do not expect readers to agree without further discussion.

14 One reviewer adds that there may be an important difference in responsibility (or culpability) in these cases. Someone who supplies an assassin with poison may not be as responsible (or culpable) for a subsequent killing as the assassin themselves. Similarly, for someone who prescribes an abortifacient, the choice—and so, responsibility—to follow through with the act lies with the patient themselves. In that regard, the prescriber may seem less responsible than the patient (where ‘responsibility’ is used in a causal sense, not a moral sense). If so, then there is yet another reason to distinguish between prescribing abortifacients and performing abortions.

15 This suggestion may cause problems for restrictive states. Recall: abortion opponents often seek to penalize abortion *providers*, rather than patients [19–21]. From an anti-abortion perspective, if patients are not to be punished, then prescribers may look like the primary wrongdoers in cases of self-administered abortion. So, it makes sense for restrictive states to focus on trying to penalize prescribers. Still, this complication yields only a partisan reason to equate acts of prescribing abortifacients with abortive acts. It seems to me there is no good nonpartisan, principled reason to collapse this distinction. And, as noted above, there are practical alternatives that would reduce confusion anyway: laws can assign harsh penalties to prescribers while also clarifying how self-administered abortions will be treated. Thus, restrictive states can solve the problems generated by self-administered abortions without denying the distinction between abortion and the enabling of abortion.

16 Additionally, if ‘abortion’ is ‘the mere termination of pregnancy,’ the term’s meaning would become so diluted as to render it useless in public policy and debate.

17 To be clear, restrictive states’ laws often describe three categories of action: abortive acts, acts that are non-abortive if performed with certain intentions, and medically necessary abortions (which are abortions, but are allowed anyway).

18 ‘Responsibility’ here is used in a causal sense, not a moral sense, so it is not synonymous with blame or guilt. If one is responsible for having performed an action, it means they are the one who performed the action; they are the primary locus from which the action arose.

19 Moreover, worrisome implications stem from claiming abortion applies only to cases of ‘known’ pregnancy. If so, then women who want to evade abortion restrictions are (in effect) encouraged to remain ignorant of their own health (e.g., by refusing to take pregnancy tests). This undermines one’s ability to make *informed* reproductive choices. Failing to diagnose pregnancy in a timely manner—and using certain abortifacients in ignorance—may also pose a serious risk to women with undetected ectopic pregnancies [63, p. 291]. So, even though abortion proponents have practical reasons to include strict knowledge requirements into definitions of ‘abortion,’ there are better reasons not to do so.

20 ‘Abortion,’ so defined, will not refer to many phenomena—such as naturally-occurring fetal demise—that are sometimes referred to by terms like ‘miscarriage’ or ‘spontaneous abortion.’ Readers who accept medical definitions of ‘abortion’ that *do* refer to naturally-occurring fetal demise as a kind of abortion may be unhappy with this implication. In response, it was demonstrated (above) that not all ‘medical definitions’ of ‘abortion’ refer to naturally-occurring fetal demise in the first place (see, especially, footnote 10). The medical community (and others) must *decide* whether to define ‘abortion’ in a way that refers to things like naturally-occurring fetal demise. As one considers how best to make this decision, it will be important to weigh the reasons for different proposals. There are clear advantages to distinguishing between ‘abortion’ and ‘miscarriage’ in public policy. First, under the proposed definition, abortion restrictions would not prevent clinicians from providing miscarriage-related care. Second, some data suggest that using language other than ‘spontaneous abortion’ to describe naturally-occurring fetal demise is favored by the vast majority of women [58, p. 278 n.3]. In addition to respecting many women’s linguistic preferences, rejecting the phrase ‘spontaneous abortion’ may also go some distance in reducing the “misplaced guilt” and “disenfranchised grief” that is commonly experienced by bereaved parents [58, pp. 274–277]. Third, insofar as a definition of ‘abortion’ should track all and only phenomena that are contentious in public debates, one *should* exclude naturally-occurring fetal demise from counting as a kind of abortion. After all, while naturally-occurring fetal demise may be regarded as tragic by abortion opponents and abortion proponents alike, it is not subject to the same kind of widespread debate as practices captured by my proposed definition of ‘abortion.’.

21 I will have much more to say about hysterotomies and hysterectomies below.

22 ‘Being’ because *e* is a human organism—an individual member of the species, *Homo sapiens*—which is not to say or imply that *e* is a person, legally or otherwise.

23 I am grateful to an anonymous reviewer for raising this point.

24 Similarly, suppose that a previable fetus is irretrievably dying. Induced delivery—or a hysterotomy or hysterectomy—would not obviously count as an abortion under those circumstances. I will unpack this point below, when describing cases in which allowing someone to die can be consistent with maintaining a real regard for their survival.

25 One could argue that, clinically speaking, mechanics and tools are all that matter when individuating between actions. This claim rests upon metaphysical assumptions about action individuation. A *philosophical* defense (rather than a ‘medical’ or ‘scientific’ defense) would be needed for that view.

26 There is much to say about distinctions between direct killing, indirect killing, and ‘letting die’ here. Some authors argue that a hysterotomy (or induced delivery prior to viability) involves the indirect killing of *e* [75, p. 114]. Others claim that these acts are not a form of killing but, instead, are merely a ‘letting die’ [68]. I need not take a stance here. As I have framed things, if someone directly and intentionally kills *e*, then they cannot claim to be acting with a real regard for *e*’s survival. Procedures that directly and intentional kill *e*, therefore, will count as abortions on my definition. That said, below, I argue that letting *e* die does not always imply that one acts without regard for *e*’s survival. By the same token, certain acts of indirect killing also seem compatible with maintaining an adequate regard for *e*’s survival. So, whether one thinks that previable delivery involves an indirect killing or merely a ‘letting die,’ there will be some cases in which the acts do not count as abortions on my definition. Lastly, suppose that someone believes that hysterotomies, hysterectomies, and previable deliveries *do* involve a direct killing of *e* (in some cases at least) [39, p. 73]. Can someone directly kill *e* (albeit *unintentionally*) while claiming to act with a real regard for *e*’s survival? Certainly. Suppose, for example, that a fetus’s survival depends upon her receiving open-heart surgery. Yet, when performing the surgery, the surgeon nicks an artery which results in the fetus’s death. The surgeon directly kills the fetus in this case, albeit unintentionally. But the surgeon was also acting with a real regard for *e*’s survival. In fact, she was trying to *save e’s* life. Thus, not every act of directly killing *e* will count as an abortion on my definition. That is, in principle, some unintentional, direct killings of *e* are non-abortive actions. Alternatively, if one wishes to argue that *every* hysterotomy, hysterectomy, and previable delivery involves the intentional, direct killing of *e*, then of course, all such acts count as abortions. But, like many other scholars and professionals, I do not find this view plausible. [38, 40] Toni Saad, for example, argues that even in extreme cases where a mother’s life is at risk, “ending a pregnancy with feticidal intention is never necessary” [76, p. 247]. If so, then whether saving the mother’s life requires direct unintentional killing, indirect unintentional killing, or merely letting *e* die, it remains possible, on my definition, that the intervention will not count as an abortion. Critically, the question of whether an act is a direct killing or not is of secondary importance anyway. What is of primary importance, in this context, is determining whether an act is performed without regard for *e*’s survival. If the act—be it a killing or letting die—secures the death of *e* and is performed without regard for *e*’s survival, then it is an abortion.

27 I would say the same thing even if one regards previable delivery (or a hysterotomy or hysterectomy) as an *indirect* killing: under certain circumstances, indirect killing will not count as an abortion, because it is possible to perform the action while maintaining one’s regard for *e*’s survival.

28 What about a case in which a pregnant woman is completely indifferent to *e*’s survival but, it just so happens, a hysterotomy must be performed at 12 weeks’ gestation to save the mother’s life? Strictly speaking, my definition might regard this act as an abortion. In practice, abortion restrictions would not preclude the action, however. This is because ending pregnancy in those circumstances is *consistent with* adequate regard for *e*’s survival. From the standpoint of public policy, if someone acts in a way that is consistent with adequate regard for *e*’s survival, then the act may be counted as a ‘non-abortion.’ In other words, if it were established that a subject opted for induced delivery prior to viability in a case where she would die were pregnancy to continue, the state might reasonably regard the act as a non-abortion (since the action is fully consistent with what someone who has sufficient regard for *e*’s survival would do).

29 I am grateful to Cody Feikles for pushing me on these points.

30 Obviously, the mother’s survival is a necessary condition for *e*’s survival prior to viability. Even if attempts to save the mother’s life risk some serious, indirect harm to *e*, therefore, the act may still be performed with regard for *e*’s survival. The act, after all, aims to actualize one of the conditions *required* for *e*’s survival.

31 Many people provided feedback during the drafting of this essay. I am grateful for comments from Bruce P. Blackshaw, Daniel Rodger, David Hershenov, Monica Snyder, Cody Feikles, two anonymous reviewers, and audiences at University at Buffalo and the 2024 American Society for Bioethics and Humanities Annual Conference.

## References

[1] Dobbs v Jackson Women’s Health Organization. 2022. 597 U.S.

[2] Carpenter, Hannah, Georgia Loutrianakis, Peyton Baker, Tiffany Bystra, and Lisa Campo-Engelstein. 2024. Procreative loss without pregnancy loss: The limitations of fetal-centric conceptions of pregnancy. *Journal of Medical Ethics* 50 (5): 310–311. 38326020 10.1136/jme-2023-109811

[3] Arey, Whitney, Klaira Lerma, Anitra Beasley, Lorie Harper, Ghazaleh Moayedi, and Kari White. 2022. A preview of the dangerous future of abortion bans – Texas senate bill 8. *New England Journal of Medicine* 387 (5): 388–390. 35731914 10.1056/NEJMp2207423

[4] Minkoff, Howard, Raaga Unmesha Vullikanti, and Mary Faith Marshall. 2024. The two front war on reproductive rights–when the right to abortion is banned, can the right to refuse obstetrical interventions be far behind? *American Journal of Bioethics* 24 (2): 11–20. 10.1080/15265161.2023.2262960 37830758

[5] Nambiar, Anjali, Shivani Patel, Patricia Santiago-Munoz, Catherine Y. Spong, and David B. Nelson. 2022. Maternal morbidity and fetal outcomes among pregnant women at 22 weeks’ gestation or less with complications in 2 Texas hospitals after legislation on abortion. *American Journal of Obstetrics & Gynecology* 227 (4): 648-650.e1. 35803323 10.1016/j.ajog.2022.06.060

[6] Colgrove, Nicholas. 2023. Unintended intrauterine death and preterm delivery: What does philosophy have to offer? *Journal of Medicine & Philosophy* 48 (3): 195–208.

[7] Hersey, Alicia E., Jai-Me. Potter-Rutledge, and Benjamin P. Brown. 2023. Abortion policies at the bedside: Incorporating an ethical framework in the analysis and development of abortion legislation. *Journal of Medical Ethics* 50 (1): 2–5. 36585243 10.1136/jme-2022-108412

[8] Räsänen, Joona, Claire Gothreau, and Kasper Lippert-Rasmussen. 2022. Does overruling Roe discriminate against women of (colour)? *Journal of Medical Ethics* 48 (12): 952–956. 36180204 10.1136/jme-2022-108504

[9] American College of Obstetricians and Gynecologists. 2022. ACOG statement on the decision in Dobbs v. Jackson. *Advocacy and Health Policy*. https://www.acog.org/news/news-releases/2022/06/acog-statement-on-the-decision-in-dobbs-v-jackson. Accessed 19 February 2024.

[10] Donley, Greer, and Caroline M. Kelly. Forthcoming. Abortion disorientation (draft only). *Duke Law Journal*. https://papers.ssrn.com/sol3/papers.cfm?abstract_id=4729217. Accessed 29 May 2024.

[11] Gallup Poll. Abortion. *In Depth Topics*. https://news.gallup.com/poll/1576/abortion.aspx. Accessed 30 December 2024.

[12] Sadler, Thomas W. 2019. *Langman’s medical embryology*, 14th ed. Philadelphia: Wolters Kluwer.

[13] Gold, Rachel B. 2005. The implications of defining when a woman is pregnant. *Guttmacher Institute* 8 (2): 7–10.

[14] Cauterucci, Christina. 2023. Birth control is next. *Slate*. https://slate.com/news-and-politics/2023/04/birth-control-is-next-republicans-abortion.html. Accessed 19 February 2024.

[15] Arizona Revised Statutes Title 36–2151. 2024.

[16] Idaho Code 18–604. 2023.

[17] Kansas Statutes 65–6702. 2024.

[18] South Carolina Code 38–71–146. 2024.

[19] Witherspoon, James S. 1985. Reexamining Roe: Nineteenth-century abortion statutes and the fourteenth amendment. *St. Mary’s Law Journal* 17 (1): 29–77. 11655872

[20] Beckwith, Francis J. 2007. *Defending life: A moral and legal case against abortion choice*. Cambridge: Cambridge University Press.

[21] Anderson, Matthew L. 2023. Anti-abortionist action theory and the asymmetry between spontaneous and induced abortions. *Journal of Medicine & Philosophy* 48 (3): 209–224. 37078978 10.1093/jmp/jhad011

[22] Andrade-Rhoades, Amanda. 2023. Abortion bans don’t prosecute pregnant people. That may be about to change. *The 19*^*th*^*News Network*. https://19thnews.org/2023/01/abortion-bans-pregnant-people-prosecution/. Accessed 20 February 2024.

[23] Overall, Christine. 2015. Rethinking abortion, ectogenesis, and fetal death. *Journal of Social Philosophy* 46 (1): 126–140.

[24] Bohn, Jessalyn A. 2022. The feminists’ dilemmas: A response to Overall’s ‘rethinking abortion, ectogenesis, and fetal death.’ In *Agency, pregnancy and persons: Essays in defense of human life*, ed. Nicholas Colgrove, Bruce P. Blackshaw, and Daniel Rodger, 195–211. New York: Routledge.

[25] Condic, Maureen L., and Donna Harrison. 2018. Treatment of ectopic pregnancy. *Linacre Quarterly* 85 (3): 241–251. 30275609 10.1177/0024363918782417 PMC6161225

[26] Buskmiller, Cara. 2024. Ectopic pregnancy as previable delivery. *Christian Bioethics* 30 (2): 120–133.

[27] Iowa Code Section 146B.1. 2024.

[28] Iowa Code Section 217.41B. 2024.

[29] Kentucky Revised Statutes 311.720. 2024.

[30] Kentucky Revised Statutes 311.732. 2024.

[31] Colorado Revised Statutes 13–22–703. 2024.

[32] Colorado Revised Statutes 25–6–402. 2024.

[33] Guttmacher Institute. 2024. U.S. abortion policies and access after *Roe*. https://states.guttmacher.org/policies/colorado/abortion-policies. Accessed 26 December 2024.

[34] Cunningham, F. Gary., Kenneth J. Leveno, Jodi S. Dashe, Barbara L. Hoffman, Catherine Y. Spong, and Brian M. Casey. 2022. *Williams Obstetrics*, 26th ed. New York: McGraw-Hill.

[35] Merriam-Webster. 2024. Abortion. *Merriam-Webster Medical Dictionary*. https://www.merriam-webster.com/dictionary/abortion#medicalDictionary. Accessed 28 December 2024.

[36] American College of Obstetricians and Gynecologists. 2018. reVITALize: gynecology data definitions. *Health IT and Clinical Informatics*. https://www.acog.org/practice-management/health-it-and-clinical-informatics/revitalize-gynecology-data-definitions. Accessed 30 December 2024.

[37] Prager, Sarah, Elizabeth Micks, and Vanessa K. Dalton. Pregnancy loss (miscarriage): terminology, risk factors, and etiology. *UpToDate*. https://www.uptodate.com/contents/pregnancy-loss-miscarriage-terminology-risk-factors-and-etiology. Accessed 30 December 2024.

[38] Yoder, Katie. 2022. Ectopic pregnancies, miscarriage: abortion ‘never necessary,’ these doctors say. *Catholic News Agency*. https://www.catholicnewsagency.com/news/251941/ectopic-pregnancies-miscarriage-abortion-never-necessary-these-doctors-say. Accessed 30 December 2024.

[39] Anderson, Marie A., Robert L. Fastiggi, David E. Hargroder, Joseph C. Howard Jr, and C. Ward Kischer. 2011. Ectopic pregnancy and Catholic morality: A response to recent arguments in favor of salpingostomy and methotrexate. *National Center for Bioethics* 11 (1): 65–82.

[40] American Association of Pro-life OBGYNs. 2022. AAPLOG responds to ‘facts are important: understanding ectopic pregnancy.’ *Integrity in Medicine*. https://aaplog.org/aaplog-responds-to-facts-are-important-understanding-ectopic-pregnancy/. Accessed 29 December 2024.

[41] National Health Service. 2024. Types of abortion. *NHS Inform*. https://www.nhsinform.scot/tests-and-treatments/non-surgical-procedures/abortion/types-of-abortion/. Accessed 30 December 2024.

[42] Callahan, Daniel. 2012. A memoir of an interdisciplinary career. In Daniel Callahan (ed.), *The Roots of Bioethics: Health, Progress, Technology, Death*. Oxford: Oxford University Press.

[43] Craddock, Joshua J. 2017. Protecting prenatal persons: Does the fourteenth amendment prohibit abortion? *Harvard Journal of Law & Public Policy* 40: 539–571.

[44] Horner, Claire, and Lisa Campo-Engelstein. 2020. Dueling definitions of abortifacient: How cultural, political, and religious values affect language in the contraception debate. *Hastings Center Report* 50 (4): 14–19. 10.1002/hast.1161 33448401

[45] American College of Gynecologists and Obstetricians. 2024. How your fetus grows during pregnancy. https://www.acog.org/womens-health/faqs/how-your-fetus-grows-during-pregnancy. Accessed 19 February 2024.

[46] National Institutes of Health. 2024. Pregnancy. https://www.nichd.nih.gov/health/topics/factsheets/pregnancy. Accessed 19 February 2024.

[47] National Health Services. 2021. You and your pregnancy at 1 to 2 weeks. https://www.nhs.uk/pregnancy/week-by-week/1-to-12/1-2-3-weeks/. Accessed 19 February 2024.

[48] Merriam-Webster. 2024. Pregnant. *Merriam-Webster Dictionary*. https://www.merriam-webster.com/dictionary/pregnant. Accessed 19 February 2024.

[49] Manne, Kate. 2020. *Entitled: How male privilege hurts women*. New York: Crown.

[50] Colgrove, Nicholas, Bruce P. Blackshaw, and Daniel Rodger, eds. 2022. *Agency, pregnancy and persons: Essays in defense of human life*. New York: Routledge.

[51] Kaczor, Christopher. 2022. The artificial womb and the end of abortion. In *Agency, pregnancy and persons: Essays in defense of human life*, ed. Nicholas Colgrove, Bruce P. Blackshaw, and Daniel Rodger, 176–194. New York: Routledge.

[52] Secular Pro-Life. 2024. A secular case against abortion. https://secularprolife.org/abortion/. Accessed 20 February 2024.

[53] New Wave Feminists. 2018. NWF mission statement. https://www.newwavefeminists.com/about. Accessed 20 February 2024.

[54] Wolf-Devine, Celia. 1989. Abortion and the ‘feminine voice.’ *Public Affairs Quarterly* 3 (3): 81–97. 11652546

[55] Mathewes-Green, Frederica. 2013. How feminism went wrong: Abortion as the price for conformity with the male model. *Christian Bioethics* 19 (2): 130–132.

[56] Bachiochi, Erika. 2021. *The rights of women: Reclaiming a lost vision*. Notre Dame, IN: Notre Dame University Press.

[57] Colgrove, Nicholas. 2024. Despite benefits of over-the-counter birth control pills, there are also risks. *The Augusta Chronicle*. https://www.augustachronicle.com/story/opinion/columns/2024/06/06/ethical-issues-to-consider-with-over-the-counter-birth-control-access/73995248007/. Accessed 30 December 2024.

[58] Bohn, Jessalyn A. 2023. When words fail: ‘miscarriage’, referential ambiguity, and psychological harm. *Journal of Medicine & Philosophy* 48 (3): 265–282. 37061800 10.1093/jmp/jhad013

[59] Lee, Ellie. 2005. Debating late abortion: Time to tell the truth. *Journal of Family Planning and Reproductive Health Care* 31 (1): 7–9. 15720839 10.1783/0000000052972933

[60] Olohan, Mary Margaret. 2023. House passes bill protecting babies born alive in botched abortions. *The Daily Signal*. https://www.dailysignal.com/2023/01/11/democrats-block-bill-protecting-babies-born-alive-in-botched-abortions/. Accessed 20 February 2024.

[61] Foster, Diana G., M. Antonia Biggs, Lauren Ralph, Caitlin Gerdts, Sarah Roberts, and M. Maria Glymour. 2018. Socioeconomic outcomes of women who receive and women who are denied wanted abortions in the United States. *American Journal of Public Health* 108 (3): 407–413. 29345993 10.2105/AJPH.2017.304247 PMC5803812

[62] Israel, Melanie. 2023. Abortion pills, coercion, and abuse. *The Heritage Foundation*. https://www.heritage.org/life/commentary/abortion-pills-coercion-and-abuse. Accessed 20 February 2024.

[63] Miller, Calum. 2022. Telemedicine abortion: Why it is not safe for women. In *Agency, pregnancy and persons: Essays in defense of human life*, ed. Nicholas Colgrove, Bruce P. Blackshaw, and Daniel Rodger, 288–300. New York: Routledge.

[64] Kaczor, Christopher. 2015. *The ethics of abortion: Women’s rights, human life, and the question of justice*, 2nd ed. New York and London: Routledge.

[65] Tollefsen, Christopher. 2015. Double effect and two hard cases in medical ethics. *American Catholic Philosophical Quarterly* 89 (3): 407–420.

[66] Hershenov, David, and Philip A. Reed. 2021. How not to defend the unborn. *Journal of Medicine & Philosophy* 46 (4): 414–430. 34219159 10.1093/jmp/jhab011

[67] Sulmasy, D.P. 1998. Killing and allowing to die: Another look. *Journal of Law, Medicine & Ethics* 26 (1): 55–64. 10.1111/j.1748-720x.1998.tb01906.x 11067586

[68] Simuklet, W. 2019. Two tragedies argument: Two mistakes. *Journal of Medical Ethics* 45 (8): 562–564. 31221765 10.1136/medethics-2019-105587

[69] Sheppard, Stephen M. (ed.). 2012. Manslaughter (manslaughterer). *Bouvier Law Dictionary: Desk Edition (Vol 2)*. Waltham, MA: Aspen Publishing. https://public.fastcase.com/Wl%2b2t%2beVuI35%2fN70vAMFZo4GW1MYLdOh49DrVbX8Op5y6narY6zTghf0f%2fOYPinIVBe5h0WAKcRN7Eo4UGsmkg%3d%3d Accessed 20 February 2024.

[70] Sheppard, Stephen M. (ed.). 2012. Recklessness (reckless). *Bouvier Law Dictionary: Desk Edition (Vol 2)*. Waltham, MA: Aspen Publishing. https://public.fastcase.com/Wl%2b2t%2beVuI35%2fN70vAMFZjJMDt8L241WMcxtC5RYovu%2bzdy5J1gqTTBR2irw8chOo%2b2bLhMa0JjUtsgISXoYkA%3d%3d Accessed 20 February 2024.

[71] American College of Obstetricians and Gynecologists. 2017. Periviable birth. *Obstetric Care Consensus 6*. https://www.acog.org/clinical/clinical-guidance/obstetric-care-consensus/articles/2017/10/periviable-birth Accessed 30 December 2024.

[72] Pizzuto, Katerina, Cory Ozimok, Radenka Bozanovic, Kathleen Tafler, Sarah Scattolon, Nicholas A. Leyland, and Michelle Morais. 2018. Hysterectomy with fetus in situ for uterine rupture at 21-week gestation due to a morbidly adherent placenta. *Case Reports in Obstetrics and Gynecology* 5430591: 1–4. 10.1155/2018/5430591 PMC6139238 30245897

[73] Miller, Calum. 2023. The scourges: Why abortion is even more morally serious than miscarriage. *Journal of Medicine & Philosophy* 48 (3): 225–242. 37061804 10.1093/jmp/jhad014 PMC10185666

[74] Thomson, Judith J. 1971. A defense of abortion. *Philosophy & Public Affairs* 1 (1): 47–66.

[75] Rhonheimer, M. 2009. *Vital conflicts in medical ethics: a virtue approach to craniotomy and tubal pregnancies*. Catholic University of America Press.

[76] Saad, T. 2022. Is abortion medically necessary? In *Agency, pregnancy and persons: Essays in defense of human life*, ed. Nicholas Colgrove, Bruce P. Blackshaw, and Daniel Rodger, 246–265. New York: Routledge.

[77] Hershenov, David. 2024. Abortion pills: Killing or letting die? *Christian Bioethics* 30 (2): 134–144.

